# Diblock Copolymers of Methacryloyloxyethyl Phosphorylcholine and Dopamine Methacrylamide: Synthesis and Real-Time Adsorption Dynamics by SEIRAS and RAIRS

**DOI:** 10.1021/acs.langmuir.3c03925

**Published:** 2024-03-08

**Authors:** Marijus Jurku̅nas, Martynas Talaikis, Vaidas Klimkevičius, Vaidas Pudžaitis, Gediminas Niaura, Ričardas Makuška

**Affiliations:** † Institute of Chemistry, Vilnius University, Naugarduko Str. 24, 03225 Vilnius, Lithuania; ‡ Department of Organic Chemistry, Center for Physical Sciences and Technology (FTMC), Sauletekio Ave. 3, 10257 Vilnius, Lithuania

## Abstract

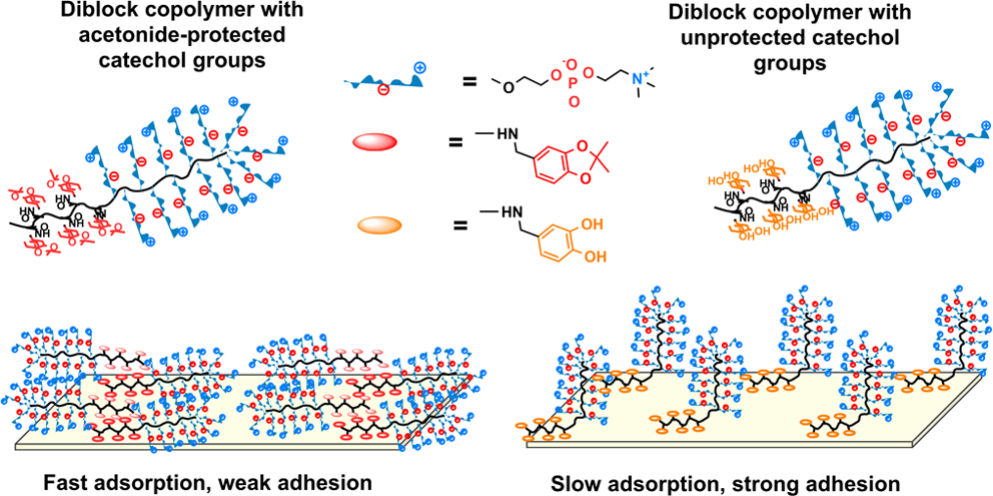

Amphiphilic diblock copolymers containing a block of 2-methacryloyloxyethyl phosphorylcholine (MPC) with unique properties to prevent nonspecific protein adsorption and enhance lubrication in aqueous media and a block of dopamine methacrylamide (DOPMA) distinguished by excellent adhesion performance were synthesized by reversible addition fragmentation chain transfer (RAFT) polymerization for the first time. The DOPMA monomer with an acetonide-protected catechol group (acetonide-protected dopamine methacrylamide (ADOPMA)) was used, allowing the prevention of undesirable side reactions during polymerization and oxidation during storage. The adsorption behavior of the diblock copolymers with protected and unprotected catechol groups on gold surfaces was probed using attenuated total reflection (ATR)-Fourier transform infrared (FTIR) spectroscopy, surface-enhanced infrared absorption spectroscopy (SEIRAS), and reflection–absorption infrared spectroscopy (RAIRS). The copolymers pMPC-*b*-pADOPMA demonstrated physisorption with rapid adsorption and ultrasound-assisted desorption, while the copolymers pMPC-*b*-DOPMA exhibited chemical adsorption with slower dynamics but a stronger interaction with the gold surface. SEIRAS and RAIRS allowed proving the reorientation of the diblock copolymers during adsorption, demonstrating the exposure of the pMPC block toward the aqueous phase.

## 1 Introduction

Biocompatible polymers can be applied in biological systems and perform their provided functions without excessive negative responses from the system. Among biocompatible polymers, a polymer family based on the monomer 2-methacryloyloxyethyl phosphorylcholine (MPC), has exclusive importance for both fundamental studies and applications.^1−4^ MPC polymers are synthetic phospholipid polymers with zwitterionic phosphorylcholine head groups, which can form cell-membrane-like structures on surfaces of various materials.^3,5^ This special surface structure has a unique property to prevent nonspecific protein adsorption and to provide efficient lubrication.

Biocompatible statistical copolymers containing MPC units were typically prepared via conventional free-radical copolymerization.^6−8^ The use of the methods of reversible-deactivation (living) radical polymerization enabled to prepare MPC copolymers of linear structure with a relatively low dispersity index, and of various architectures, such as block-type, graft-type, and gradient copolymers.^9−13^ Despite extensive studies on the development of novel functional copolymers containing MPC units, the most challenging aspect has been the efficient immobilization of such copolymers onto the surfaces of various substrates.

Two strategies are known to immobilize polymers onto surfaces, scilicet, “grafting to” via physical adsorption and “grafting from” via surface-initiated polymerization. Surface-initiated living radical polymerization of MPC was demonstrated to form polymer brushes with unique properties arising from the high density of polymer graft chains.^14−16^ “Grafting from” is preferential in many cases, giving a chemically bonded layer with a high graft density. However, this approach usually is more complicated since it involves the pretreatment of chemically inert surfaces to create active sites initiating or controlling chemical grafting reactions. In turn, “grafting to” is more convenient from the practical point of view, providing an opportunity to synthesize particular polymers in advance and decorate surfaces of complex configuration in a simple manner. However, for effective surface modification using a “grafting to” approach, the polymers must possess suitable anchoring groups.

In recent years, there has been significant interest in biomimetic adhesives inspired by mussel adhesive proteins.^17−20^ The excellent adhesion of mussels on a wide range of surfaces and items in wet conditions is determined by catechol-containing amino acid 3,4-dihydroxyphenyl-l-alanine (l-DOPA). To imitate the remarkable adhesive properties of these proteins, a number of synthetic polymers containing the catechol group were synthesized and studied.^21,22^ The wide range of polymers with catechol groups provide a fast and efficient method for applying coatings by “grafting to” on surfaces of various origins. A simple dip of the substrate into the polymer solution is often enough to cover the protection layer.

One of the most widely used monomers containing a catechol moiety is dopamine methacrylamide (DOPMA). DOPMA was copolymerized with many commercially available monomers.^23−30^ Diblock copolymers were obtained by the successive reversible addition fragmentation chain transfer (RAFT) polymerization of DOPMA and dimethylacrylamide.^31^ The RAFT copolymerization of acetonide-protected DOPMA yielded diblock copolymers with dopamine acrylamide^32^ and statistical copolymers with poly(ethylene oxide) methyl ether methacrylate (PEOMEMA).^33^ It was demonstrated^34,35^ that the underwater wear resistance of the adsorbed layers is high in the case of statistical catechol copolymers p(DOPMA-*co*-PEOMEMA), and it is exclusively high in the case of diblock copolymers pDOPMA-*b*-pPEOMEMA.

During the last years, statistical copolymers of MPC and DOPMA were synthesized by conventional free-radical copolymerization.^36−42^ The copolymers p(MPC-*co*-DOPMA) with both adhesion property and antifouling property were used for fabrication of the lubricating microspheres,^38^ modification of the surface of biodegradable mesoporous silica nanoparticles to prepare dual-functional nanoparticles,^39^ modification of the titanium alloy for enhanced lubrication and bacterial resistance,^40^ and preparation of efficient scavengers of reactive oxygen species.^41^ One should note that statistical copolymers of MPC and DOPMA were used in all of the cases. To the best of our knowledge, there are no reports in the scientific literature concerning the synthesis and application of the diblock copolymers of MPC and DOPMA.

Infrared spectroscopy, encompassing techniques such as attenuated total reflection (ATR), surface-enhanced infrared absorption spectroscopy (SEIRAS), reflection–absorption infrared spectroscopy (RAIRS), etc. provides detailed information about molecular structures, orientations, and interactions at surfaces and interfaces. SEIRAS offers enhanced sensitivity to the molecular structure and orientation on solid surfaces, attributed to the metal surface plasmon resonance effect. Such enhancement is required for detecting minor conformational changes in molecules adsorbed on the surface or within a 5–10 nm range from it. This method’s distinct advantage is its capacity to examine molecular systems *in situ* within aqueous environments and under controlled electric potentials, achieving temporal resolution down to several seconds. SEIRAS has been effectively used to investigate the adsorption and specialized functionalization of surfaces with various polymers such as poly(vinylpyrrolidone), poly(ethylene glycols), polyoxamers, polyoxamines, and others.^43−45^ A novel approach to plasmon-enhanced ultrasensitive infrared spectroscopy involves the development of plasmonic nanoantennas.^46,47^

In contrast, RAIRS is suitable for applications not demanding *in situ* analysis. RAIRS sensitivity extends beyond surface proximity, probing significantly deeper into the bulk, compared to SEIRAS. The method analyzes molecular orientations on gold surfaces based on surface selection rules, providing a deeper understanding of the behavior of surface-active segments. Together, these techniques offer key insights into the interface behavior of the copolymers, which is important for biocompatible and antifouling applications.

In the present study, inspired by the excellent adhesion performance of mussels and the superior lubrication performance of phospholipids, biomimetic diblock copolymers of 2-methacryloyloxyethyl phosphorylcholine and dopamine methacrylamide were synthesized via RAFT polymerization for the first time. The DOPMA monomer with a protected catechol group was used for the synthesis, allowing the prevention of undesirable side reactions during polymerization and preserving the synthesized copolymers from oxidation during storage. The adsorption dynamics of the diblock copolymers with acetonide-protected and unprotected catechol groups on the gold surface was studied using various infrared absorption spectroscopic techniques (attenuated total reflection (ATR)-Fourier transform infrared (FTIR) spectroscopy, SEIRAS, RAIRS), demonstrating the reorientation of the diblock copolymers at the surface and exposing pMPC blocks into the bulk.

## 2 Materials and Methods

### 2.1 Materials

Methanol (MeOH, >99.9%, Honeywell), ethyl acetate (EtOAc, >99.5%, Honeywell), *n*-hexane (Hex, >99.5%, Eurochemicals), *N*,*N*-dimethylformamide (DMF, >99.8%, Honeywell), 4,4-azobis(4-cyanovaleric acid) (ACVA, 98%, Fluka), 2-methacryloyloxyethyl phosphorylcholine (98%, Sigma-Aldrich), and trifluoroacetic acid (TFA, 99.9%, Sigma-Aldrich) were used as received. Deuterated chloroform-*d*_1_, dimethyl sulfoxide (DMSO)-*d*_6_, and methanol-*d*_4_ were purchased from DeuteroGmbH. The RAFT chain transfer agent 4-(((butylthio)carbonothioyl)thio)-4-cyanopentanoic acid (CTA) and acetonide-protected dopamine methacrylamide (ADOPMA) were synthesized according to previously published procedures.^13,33^ For the detailed synthesis procedures, please refer to the Supporting Information (SI).

### 2.2 RAFT Polymerization of MPC

The polymerization of 2-methacryloyloxyethyl phosphorylcholine (MPC) was carried out in a mixture of MeOH/H_2_O (75:25 v/v). The monomer concentration in the solution was set to 20% and the initial molar ratio of CTA to the initiator was maintained constant ([CTA]_0_/[I]_0_ = 3:1) for all polymerizations. The polymerization procedure of MPC at a molar ratio to CTA equal to 40 is presented below: MPC (1.476 g, 5.0 mmol), RAFT CTA (29.1 mg, 0.01 mmol), and ACVA (9.34 mg, 0.0033 mmol) were placed in a 25 mL round-bottom flask and dissolved in a mixture of 5.17 mL of MeOH and 1.85 mL of deionized water. The solution was bubbled for 30 min with nitrogen gas and then stirred for 8 h at 70 °C. The polymerization was stopped by quenching the flask to liquid nitrogen. The polymer was precipitated by pouring the reaction mixture into 10-fold excess of acetone and purified by reprecipitation from methanol, giving the yellowish-white powder (practical yield 85%).

### 2.3 RAFT Polymerization of ADOPMA

The polymerization of acetonide-protected dopamine methacrylamide (ADOPMA) was carried out in DMF. The monomer concentration in the solution was set to 20%, and the ratio of the monomer to CTA and the initiator [M]_0_/[CTA]_0_/[I]_0_ was equal to 500:5:1. The polymerization procedure of ADOPMA is as follows: ADOPMA (2.00 g, 7.65 mmol), RAFT CTA (22.3 mg, 0.077 mmol), and ACVA (4.29 mg, 0.015 mmol) were placed in a 25 mL round-bottom flask and dissolved in 8.47 mL of DMF. The solution was bubbled for 30 min with nitrogen gas and then stirred for 24 h at 75 °C. The polymerization was stopped by quenching the flask to liquid nitrogen. The polymer was precipitated by pouring the reaction mixture to 10-fold excess of a hexane/EtOAc mixture (8:2 v/v) and purified by reprecipitation from EtOAc to 9-fold excess of hexane, giving the yellowish-white powder (practical yield 70%, *M*_n_ 19.1 kDa, *Đ* 1.15, DP 73).

pDOPMA with unprotected catechol groups was obtained from pADOPMA via the acetonide-removing procedure as described elsewhere.^38^ The detailed description of the procedure is provided in the Supporting Information (SI). Both homopolymers pADOPMA with acetonide-protected catechol groups and pDOPMA with unprotected catechol groups were used as reference materials for vibrational marker bands in adsorption experiments.

### 2.4 Synthesis of the Diblock Copolymers pMPC-*b*-pADOPMA

Diblock copolymers pMPC-*b*-pADOPMA were synthesized by the chain extension of pMPC containing terminal trithiogroups. An example of the chain extension of pMPC, which acts as macroCTA by the units of ADOPMA keeping the ratio [M]_0_/[pMPC]_0_/[I]_0_ = 120:3:1, is presented below. 0.50 g of dry pMPC (*M*_n_ 19 000 g/mol according to size exclusion chromatography (SEC), DP 34, 0.049 mmol) was placed into a 25 mL round-bottom flask containing 10.2 mL of methanol, and the solution was stirred for an hour until full homogenization. Then, 4.66 mg of the initiator ACVA (0.0166 mmol) and 0.52 g of the monomer ADOPMA (2.0 mmol) were added to the reaction mixture. The solution was bubbled for 30 min with nitrogen gas and then stirred for 24 h at 70 °C. The polymerization was stopped by quenching the flask to liquid nitrogen, and the product was precipitated by pouring the reaction mixture to 10-fold excess of acetone and purified by reprecipitation from methanol to a mixture of hexane/EtOAc (9:1 v/v), giving the white powder (practical yield 63%).

### 2.5 Analysis and Characterization of the Copolymers

The number-average molecular weight (*M*_n_) and dispersity index (*Đ* = *M*_w_/*M*_n_) of the synthesized polymers were determined by size exclusion chromatography (SEC) using the Viscotek TDAmax (Malvern Panalytical, U.K.) system equipped with a triple detection array (TDA305) consisting of a refractive index (RI) detector, a light scattering detector (LS) simultaneously measuring the scattered light (laser 3 mW, 670 nm) at two angles, right-angle (90°) and low-angle (7°), and a four-capillary bridge viscosity detector (DP) plus a Viscotek UV detector 2500 (UV). SEC measurements of pMPC solutions were carried out in MeOH/H_2_O (3:1 v/v) as an eluent at 30 °C using a constant flow rate of 0.5 mL/min and a Viscotek A6000 M General Mixed column, 300 × 8.0 mm^2^. SEC measurements of pADOPMA solutions were carried out in DMF as an eluent at 50 °C using a constant flow rate of 1.0 mL/min and a column Viscotek T6000 M General Mixed styrene–divinylbenzene type, 300 × 8.0 mm^2^.

^1^H and ^13^C NMR spectra of pMPC, pADOPMA, and pMPC-*b*-pADOPMA and pMPC-*b*-pDOPMA were recorded on a Bruker 400 Ascend (Germany) nuclear magnetic resonance spectrometer (400 MHz) in D_2_O or MeOD-*d*_4_ at 22 °C.

Dynamic light scattering (DLS) measurements of the copolymer solutions were carried out using a Zetasizer Nano ZS (Malvern Panalytical, U.K.) equipped with a 4 mW HeNe laser at a wavelength of 633 nm. Measurements of the intensity of the scattered light were performed at an angle of 173°. The size distribution data were analyzed using Zetasizer software (v.7.12). The copolymer concentration in methanol or water was kept at 1 mg/mL.

The hydrophilicity of the copolymers was studied by measuring the water contact angle (WCA) on the copolymer layer using a Theta Lite optical tensiometer from Biolin Scientific (Finland). The WCA was measured in three different spots on the copolymer-coated gold surface. A drop of deionized water (10 μL) was applied onto the copolymer layer, and after 30 s, the picture of the drop was captured and analyzed.

### 2.6 Attenuated Total Reflectance Infrared Absorption Spectroscopy (ATR-FTIR)

Infrared spectra of bulk compounds were collected using an infrared absorption spectrometer Alpha (Bruker, Inc., Germany) equipped with a room-temperature DLATGS detector and ATR accessory (Platinum ATR Diamond). Spectra were collected with 4 cm^–1^ resolution from 128 interferogram scans.

### 2.7 Reflection–Absorption Infrared Spectroscopy (RAIRS)

The copolymer desorption from the gold surface was studied using reflection–absorption infrared spectroscopy (RAIRS) and contact angle measurements. For RAIRS, microscopic 25 × 75 mm^2^ glass slides were coated with a 2 nm Cr adhesion layer and a 150 nm gold layer using a PVD75 (Kurt J. Lesker Co.) magnetron sputtering system. Immediately after coating, the slides were submerged in aqueous copolymer solutions (0.1 mg/mL, adjusted to pH 4 using H_2_SO_4_) for 85 min. In order to desorb copolymers, slides were subjected to ultrasound in a methanol solution for 15 min. RAIRS spectra were collected before and after desorption using a Vertex 80v spectrometer (Bruker, Inc., Germany). The spectrometer was equipped with a liquid-nitrogen-cooled MCT narrow band detector and a horizontal RAIRS accessory reflecting p-polarized light at a grazing 80° angle. Spectra were collected with 4 cm^–1^ resolution and an aperture of 4 mm from 512 interferogram scans. The spectrometer and the sample compartment were evacuated (∼2 mbar) during the measurements. The spectrum of a hexadecanethiol-*d*_33_ monolayer adsorbed on gold was used as a reference. Contact angle measurements were performed by measuring the contact angle of a sessile drop. An ultrapure water drop (1–2 μL) was analyzed by the droplet shape analyzer DSA 100 (Kruss GmbH Hamburg, Germany).

### 2.8 Surface-Enhanced Infrared Absorption Spectroscopy (SEIRAS)

SEIRAS experimental details were described elsewhere.^48^ In short, the surface of the mechanically polished face-angled Si crystal was subjected to 2% HF for 1 min and then coated with gold by applying a plating mixture for 4 min at room temperature. In order to increase the mechanical resistance of the gold film, the film was removed with aqua regia, and the gold film plating was repeated.^49^ The gold plating solution consisted of equal volumes of (1) 0.15 M Na_2_SO_3_, 0.05 M Na_2_S_2_O_3_, and 0.05 M NH_4_Cl, (2) 20 wt % NH_4_F, (3) 2 wt % HF, and (4) 0.03 M NaAuCl_4_. After formation, the gold film was cleaned and activated by cyclic voltammetry (CV) in a N_2_-purged pH 5.8 sodium acetate solution (0.1 M). CV was performed using a PGSTAT101 potentiostat (Metrohm) starting within the 0–300 mV potential range vs the Ag/AgCl reference electrode. The upper potential limit was increased by 100 mV every three cycles until the onset of gold oxidation (at approximately 1.0 V). The CV scan speed was 20 mV/s. SEIRAS measurements were carried out using a spectrometer Vertex 80v (Bruker, Germany) equipped with a liquid-nitrogen-cooled MCT detector. The spectral resolution was 4 cm^–1^, the aperture was 2 mm, and 50 sample scans and 100 background scans were coadded. The SEIRAS substrate was assembled into a VeeMax III accessory with a Jackfish cell J1F (Pike Technologies), and the incident angle for an ATR unit was set to 63°. The spectrometer was purged with dry air for 15 h before measurements.

### 2.9 Theoretical Modeling

Theoretical modeling of the DOPMA fragment and the DOPMA fragment with an Au_3_ cluster was performed using Gaussian 09 for Windows.^50^ Geometry optimization and vibrational frequency calculations were performed by using the density functional theory (DFT) method and the B3LYP functional. Calculations were accomplished using the 6-311++G(2d,p) basis set for C, H, and O atoms and LANL2DZ with ECP for the gold atoms. The cluster model built from three gold atoms represents the metal surface. Calculated vibrational frequencies and intensities were scaled according to the method described elsewhere.^51^

## 3 Results and Discussion

### 3.1 Synthesis of the Diblock Copolymers pMPC-*b*-pDOPMA

**Scheme 1:**
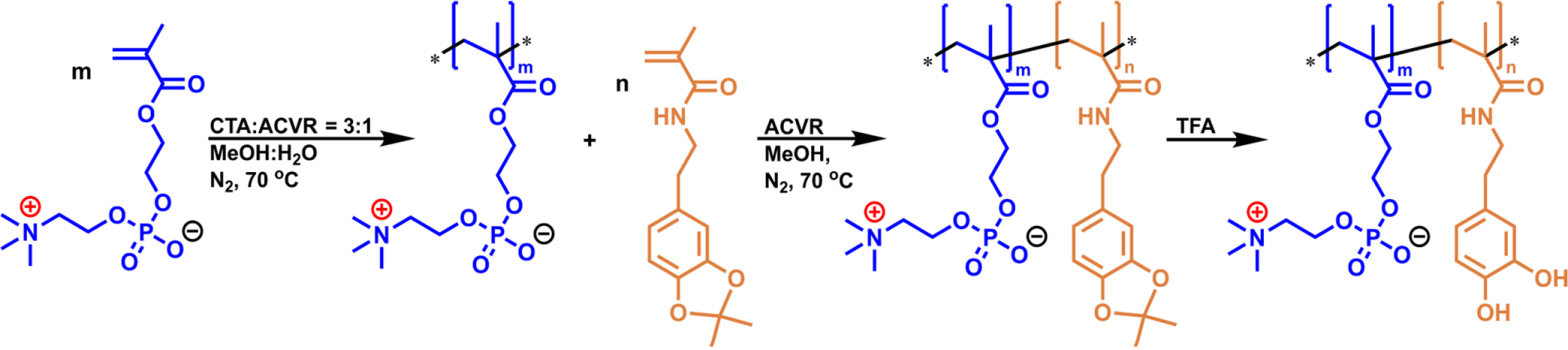
Synthesis Scheme of the Diblock Copolymers pMPC-*b*-pDOPMA

Synthetic routes to the diblock copolymers with acetonide-protected catechol groups pMPC-*b*-pADOPMA and their counterparts with unprotected catechol groups pMPC-*b*-pDOPMA are presented in Scheme 1.

**Figure 1:**
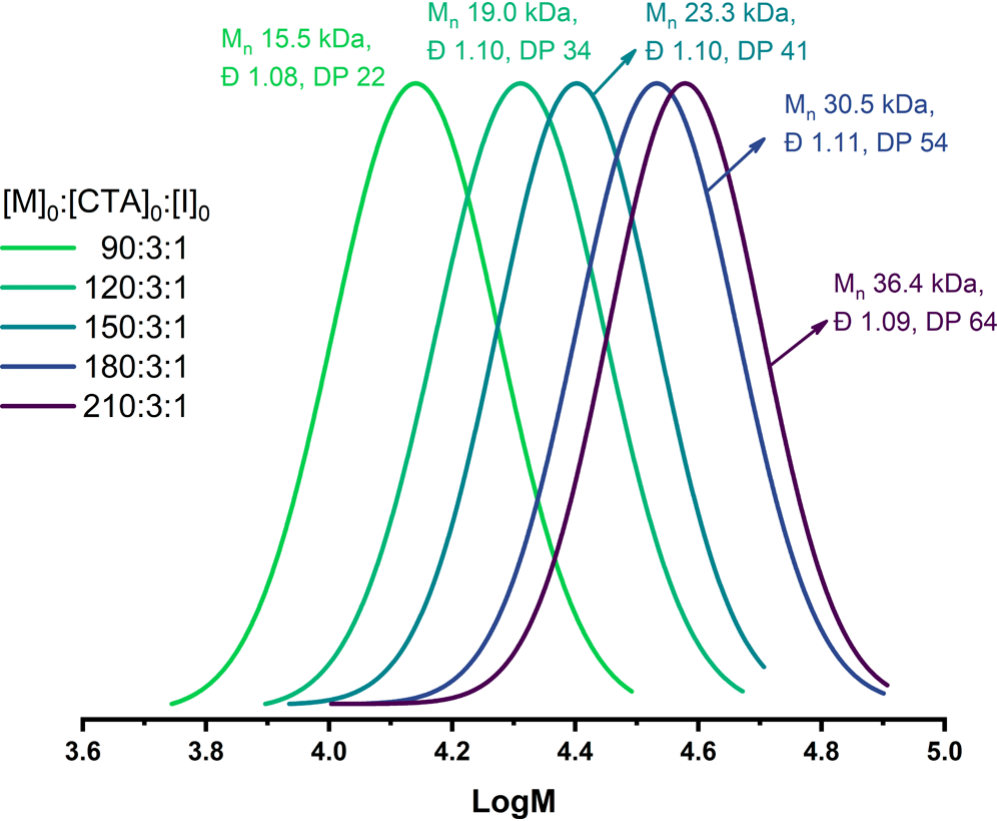
Molecular weight distribution (MWD) curves of various pMPC samples.

The polymerization of MPC has been extensively studied before.^11,12,52,53^ It was usually conducted in organic solvents or mixtures of water and water-miscible organic solvents such as methanol and other alcohols.^54,55^ In the present study, MPC was polymerized in the mixed solvent MeOH/water = 3:1 v/v. Such a mixture of the solvents was chosen adopting the polymerization mixture to direct SEC measurements.^56^ The polymerization of MPC was well controllable, giving polymers with a very low dispersity index, *Đ*, of about 1.1 (Figure 1).

It was shown previously^13^ that SEC with triple detection gave an enlarged molecular weight of pMPC because of 15 molecules of water being an integral part of the monomer. Thus, the DP of pMPC was calculated assuming that the molecular weight of the monomer is 565 (MPC + 15 molecules of H_2_O). The experimental DP of pMPC was in good agreement with that calculated theoretically taking into account the ratio of the monomer to CTA and the monomer conversion (polymer yield).

**Figure 2:**
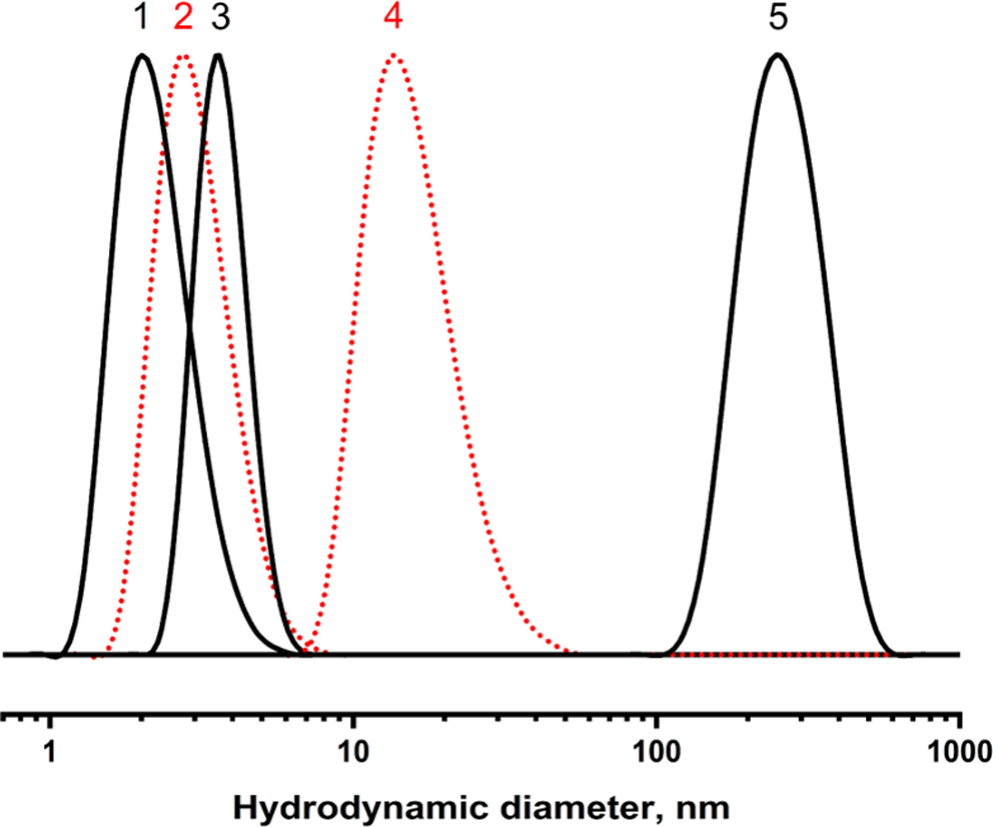
Particle distribution curves of pMPC (1, 2) and of the acetonide-protected copolymer pMPC-*b*-pADOPMA (3–5) in methanol (1, 3, 5) and water (2, 4) at 25 °C. The copolymer concentration in methanol is 1 mg/mL (3) and 50 mg/mL (5).

For the synthesis of the diblock copolymers, low-dispersity pMPC (*Đ* = 1.10) was used as the first block (macroCTA), and the ratio of [ADOPMA]/[macroCTA] was kept constant at 40:1. In previous studies, many amphiphilic diblock copolymers were synthesized starting from the pMPC block.^10,57−60^ Several solvents were used for the chain extension reaction, including methanol,^10^ ethanol,^60^ ethanol/chloroform (3:7 v/v),^58^ methanol/DMSO (1:1 v/v),^59^ and ethanol/tetrahydrofuran (THF) (1:1 or 3:1 v/v).^6^ In the present study, chain extension by the units of ADOPMA from pMPC was done in MeOH, EtOH, and several mixtures of these alcohols with DMF and CHCl_3_ (Table 1). In all of the cases, the pADOPMA block was short, with a DP not more than 13. The use of cosolvents DMF and CHCl_3_ was not justified since these solvents do not solubilize pMPC, especially with a higher DP. Methanol proved to be the most suitable solvent well solubilizing the pMPC block and partially solubilizing pADOPMA. It was shown by DLS analysis (Figure 2) that at the concentration 1 mg/mL, the block copolymers are well soluble in methanol but form aggregates at the concentration 50 mg/mL, which is similar to that in the reaction mixture during chain extension. We suppose that the insufficient solubilization of the pADOPMA block by MeOH was the main reason impeding receiving that block with a higher DP. On the other hand, such a length of the polymeric block responsible for the adhesive properties should be sufficient and contribute to achieving a higher density of the adsorbed chains.

**Table 1:** Characteristics of the Diblock Copolymers pMPC-*b*-pADOPMA

entry	solvent	copolymer composition, MPC mol %	DP1, first block (SEC)	DP2, second block (NMR)	*M*_n_, kDa
1	MeOH	72.0	22	9	8.7
2	MeOH/DMF (1:2 v/v)	67.9	22	10	9.2
3	EtOH	85.0	34	6	11.6
4	EtOH/CHCl_3_ (3:7 v/v)	82.7	34	7	11.9
5	MeOH	83.5	34	7	11.8
6	MeOH	75.9	41	13	15.3
7	MeOH	85.6	54	9	18.3
8	MeOH	86.3	64	10	21.5

Usually, chain extension by units of another monomer during the synthesis of diblock copolymers is evaluated by SEC analysis. Unfortunately, we had no possibility to study the diblock copolymers of pMPC and pADOPMA by SEC because of the absence of an eluent dissolving both blocks of a very different nature. The only common solvent for both the hydrophilic pMPC block and the hydrophobic pADOPMA block was methanol, which is not compatible with the known SEC columns. The composition of the diblock copolymers and the DP of the second block were calculated from the ^1^H NMR spectra of the copolymers taking into account the DP of the first block determined by SEC.

**Figure 3:**
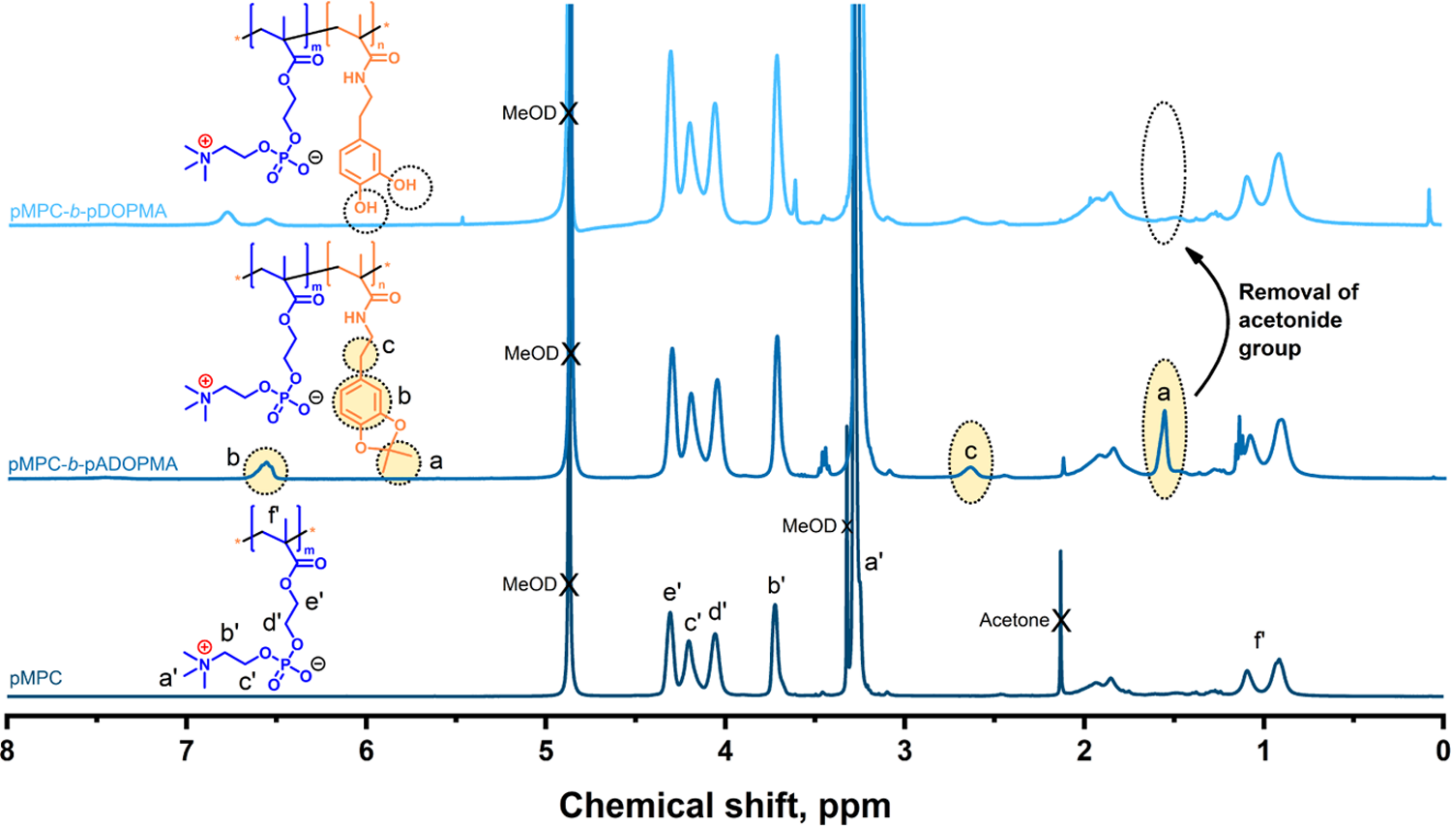
^1^H NMR spectra of pMPC, the diblock copolymer with acetonide-protected catechol groups pMPC-*b*-pADOPMA, and the diblock copolymer with unprotected catechol groups pMPC-*b*-pDOPMA in MeOD-*d*_4_.

The ^1^H NMR spectra of pMPC and the diblock copolymers pMPC-*b*-pADOPMA and pMPC-*b*-pDOPMA are presented in Figure 3. In the spectrum of pMPC, chemical shifts at 3.25 3.75, 4.05, 4.19, and 4.30 ppm are attributed to the protons in −N^+^(CH_3_)_3_, −N–CH_2_–, −O–CH_2_–CH_2_–O–P–, −N^+^–CH_2_–CH_2_–, and −COO–CH_2_–CH_2_– groups, respectively. In the spectrum of the diblock copolymer with protected catechol groups, there are three additional chemical shifts at 1.56 2.61, and 6.50 ppm (marked yellow), which are assigned to the acetonide group −C(CH_3_)_2_, the methylene group −NH–CH_2_–CH_2_–, and the benzene ring (3H), respectively.

The copolymer composition was calculated by comparing the intensity of the chemical shifts of −N–CH_2_– (2H, 3.72 ppm) and O–CH_2_–CH_2_–O–P–CH_2_– (6H, 4.6–4.0 ppm) groups of pMPC, and the intensity of the chemical shifts of acetonide (6H, 1.56 ppm) and −NH–CH_2_–CH_2_– (2H, 2.61 ppm) groups of ADOPMA. The strong chemical shift at 3.25 ppm attributed to the protons in the −N^+^(CH_3_)_3_ group of MPC was superimposed by two chemical shifts of MeOD-*d*_4_ and ADOPMA (−NH–CH_2_−), and it was not useful for quantification. The chemical shift at 6.5 ppm attributed to the aromatic protons of ADOPMA was not used for the calculation of the copolymer composition since the integral area of that chemical shift was reduced compared to other chemical shifts of ADOPMA. This could be due to the partial masking of the catechol group because of insufficient solvation by methanol. The chemical composition of the diblock copolymers determined by ^1^H NMR spectroscopy was verified by an independent method based on UV–vis adsorption spectra of the copolymer solutions in methanol (see Figures S7–S9 in the SI). Calculations based on NMR spectra and UV–vis spectra of the copolymer solutions gave close results (deviation less than 1%) (Tables S1 and S2 in the SI). The composition of the diblock copolymers served for the calculation of the DP of the second block of the copolymers (Table 1).

Trying to substantiate the presence of diblock copolymers, ^1^H NMR and ^13^C NMR spectra of the diblock copolymers pMPC-*b*-pADOPMA were recorded in D_2_O, MeOD, and the mixture of these solvents MeOD/D_2_O (1:1 v/v) (Figures S10 and S11 in the SI). The typical ADOPMA chemical shifts were hardly visible in D_2_O, while they appeared in the solvent mixture and had a maximal intensity in MeOD. Such a behavior is characteristic for amphiphilic diblock copolymers in solutions with the preferential solvation of one block.^61,62^ To ascertain the possible micellization of the diblock copolymers in aqueous solutions, ^1^H NMR spectra of the diblock copolymers in D_2_O solutions at various concentrations were recorded (Figure S12 in the the SI). These spectra were then used to calculate the DOPMA (ADOPMA) content in the copolymers (Table S3 in the SI). It was found that the DOPMA (ADOPMA) content calculated from the spectra at a concentration of 10 mg/mL of the copolymers was approximately half as much as that calculated from the spectra at a concentration of 0.1 mg/mL of the copolymers. Moreover, the calculated ADOPMA content was 3–4 times lower compared to the DOPMA content. It is evident that at a higher concentration of the copolymers, a significant portion of the catechol groups is hindered, as the monomeric units containing catechol groups are situated in the core part of the micelles. The tendency for micellization is much higher for the copolymers with acetonide-protected catechol groups. The extent of micellization of the copolymer with unprotected catechol groups, pMPC-*b*-pDOPMA, at a low concentration (0.1 mg/mL) is low since the DOPMA content calculated from the ^1^H NMR spectrum in D_2_O differs only slightly from that calculated from the ^1^H NMR spectrum in MeOD-*d*_4_, where the copolymer is fully solubilized (Table S3 in the SI).

Particle size distribution (PSD) curves of pMPC and of the block copolymer pMPC-*b*-pADOPMA in water and methanol are presented in Figure 2. pMPC is fully soluble in water and in methanol with a particle size less than 5 nm. pADOPMA is not soluble in water and is only partially soluble in methanol (short-length polymers only, not shown in Figure 2). The particle size of the diblock copolymer in water is much larger with an average value of about 20 nm; moreover, the aqueous solution of the diblock copolymer does not contain any traces of the particles with a diameter less than 5 nm. The diblock copolymer was soluble in methanol but an average value of the particle diameter was evidently larger compared to that of pMPC. These data prove the formation of the diblock copolymer with no residual (unreacted) pMPC and the side product pADOPMA.

Diblock copolymers with unprotected catechol groups pMPC-*b*-pDOPMA were obtained by the removal of acetonide protective groups. ^1^H NMR spectra confirmed the full deprotection of catechol moieties with no side products (Figure 3, the disappearance of the acetonide −C(CH_3_)_2_ signal at 1.5 ppm).

### 3.2 Dynamics of Adsorption of the Diblock Copolymers pMPC-*b*-pADOPMA and pMPC-*b*-pDOPMA on the Gold Surface Studied by the Methods of Vibrational Spectroscopy

#### 3.2.1 Vibrational Marker Bands of the Monomeric Units

**Figure 4:**
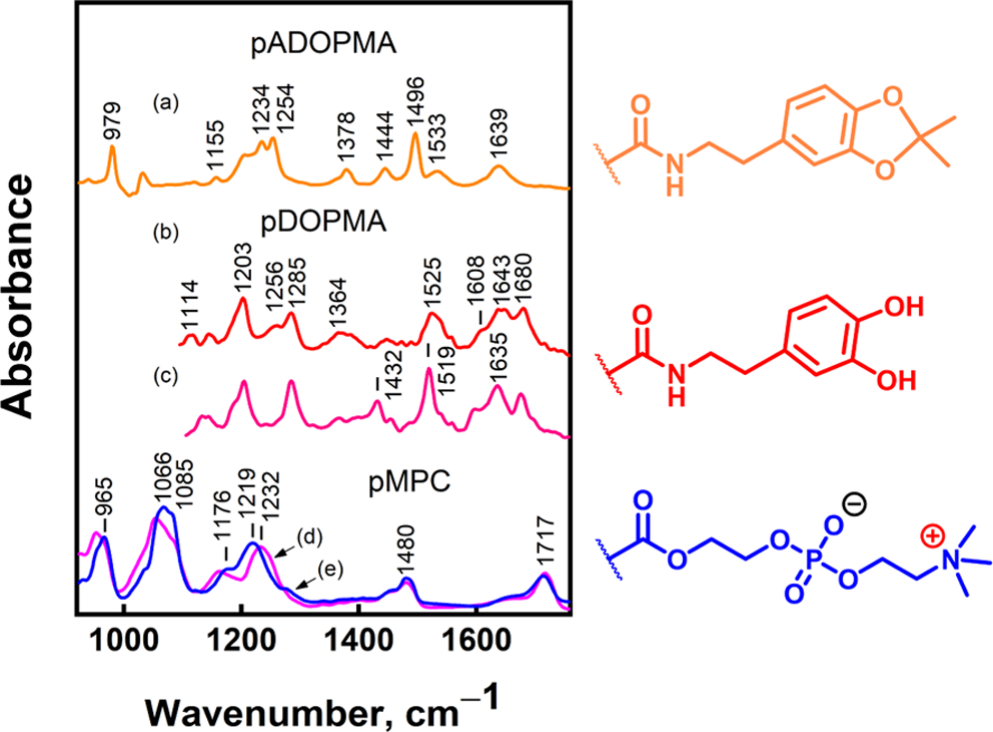
ATR-FTIR spectra of pADOPMA (*M*_n_ 19.1 kDa, *Đ* 1.15, DP 73) dissolved in (a) CH_3_OH, pDOPMA in (b) CH_3_OH and (c) CH_3_OD, (d) powder pMPC (*M*_n_ 23 300, *Đ* 1.10, DP 41), and (e) pMPC dissolved in water. The solvent spectra are subtracted. At the right, top to bottom, are the fragments of acetonide-protected dopamine in pADOPMA, dopamine in pDOPMA, and phosphorylcholine in pMPC.

The adsorption behavior of the diblock copolymers pMPC-*b*-pDOPMA and pMPC-*b*-pADOPMA on the gold surface was investigated using infrared absorption spectroscopy. Vibrational marker bands specific to acetonide-protected and -deprotected dopamine methacrylamide were determined using ATR-FTIR spectra of the methanol-dissolved homopolymers pDOPMA and pADOPMA (Figure 4). The assignment is based on literature,^63−72^ the H/D exchange experiment, and our DFT calculations (Table 2).

**Table 2:** Assignment of Spectral Bands of pADOPMA, pDOPMA, and pMPC^a^

pADOPMA		pDOPMA						pMPC
FTIR	DFT	FTIR (deut.)	DFT (deut.)	ref	assignment	FTIR	ref	assignment
979	990			(63)	ν(C–O) acetonide	965		ν_s_(N^+^(CH_3_)_3_)
		1114	1120	(64,65)	δ(=C–H), ν(C=C)	1066		ν(C–O) from ester
1204						1085 sh	(66,67)	ν_s_(PO_2_)
		1203 (1203)	1206 (964)			1176		ν(C–O)
1234	1248			(68−70)	ν(C–O) aryl	1219	(66,67)	ν_as_(PO_2_)
	1255							
n.i.		1256 (n.d.)			amide-III	1445		δ(CH_2_)
1254	1272	1285 (1285)	1294 (1292)	(68)	ν(C=C) ν_9_ + ν(C–O)	1480		δ_as_(CH_3_)
		1364	1382 (990)	(69)	δ(COH)	1717	(71)	ν(C=O)
1378	1394			(72)	δ_s_(CH_3_) gem-dimethyl			
	1406							
1444		1444			ν(C=C) ν_19a_ + δ(CH_2_)			
1496	1511	1525 (1519)	1540 (1532)	(64,65,70)	ν(C=C) ν_19b_ + ν(C–O)			
1533		1540 (1432)			amide-II			
1608	1618	1608	1624	(65)	ν(C=C) ν_8_			
	1638		1633					
1639		1643			amide-I			
		1680						

a n.i., not identified; n.d., not detected.

There is disagreement regarding the assignment of the 1496 cm^–1^ mode in dopamine derivatives in the literature. Several studies have linked this mode to the deformation motion of C–H in the gem-dimethyl group of acetonide.^69,73^ Others assigned it to the ring’s double bond stretching coupled with ν(C–O).^64,65,70^ Our DFT calculations suggest that the latter assignment is more likely. In agreement, the deprotection of catechol leads to a frequency upshift by 29 cm^–1^ to 1525 cm^–1^. This mode downshifts by 6 cm^–1^ due to the exchange of labile hydrogens to deuterons in methanol-*d* solution, which clearly evidences an involvement from C–O(H) motion to the spectral band. Such a downshift is consistent with DFT calculations (Table 2). A weak and broad band near 1364 cm^–1^ in the pDOPMA spectrum is associated with the deformation motion of catechol δ(COH); the band is not visible in the spectrum upon deuteration. The 1254 cm^–1^ mode in the pADOPMA spectrum is associated with ν(C=C) and ν(C–O), and it shifts by 31 cm^–1^ to 1285 cm^–1^ due to the deprotection of catechol. DFT predicts a similar shift, δ = 22 cm^–1^. The amide group is involved in amide-I, -II, and -III vibrations, which appear in the 1600–1700 cm^–1^ region, around 1530 cm^–1^ (or 1430 cm^–1^ upon H exchange to D), and around 1256 cm^–1^, respectively.^74^

The pMPC unit comprises a phosphorylcholine head group, whose characteristic spectral bands at 1080 and 1230 cm^–1^ are assigned to the symmetric and asymmetric phosphate group stretching, respectively. The asymmetric stretching ν_as_(PO_2_) is highly sensitive to the hydration level and may vary as much as 30 cm^–1^ in frequency; notice the band position of solid pMPC at 1232 cm^–1^ and the dissolved sample at 1219 cm^–1^, as shown in Figure 4d,e, respectively.^66,67^ The compound’s ester group gives rise to a characteristic C=O stretching vibration band ν(C=O) near 1717 cm^–1^.

#### 3.2.2 *In Situ* Monitoring of Copolymer Adsorption on the Gold Surface

**Figure 5:**
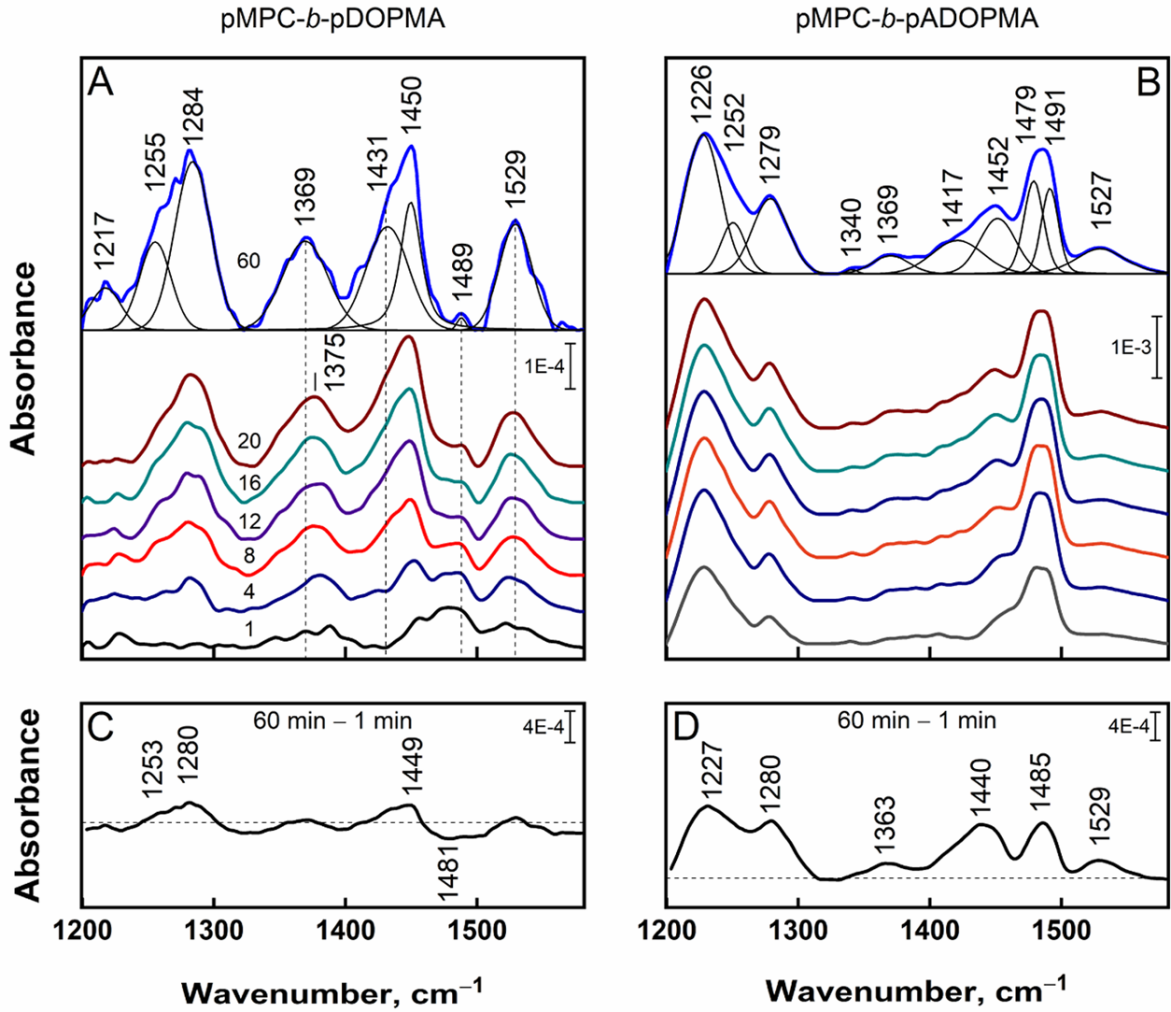
(A, B) Time-dependent SEIRAS spectra of the diblock copolymers pMPC-*b*-pDOPMA and pMPC-*b*-pADOPMA adsorbed to the gold surface in H_2_O. Incubation time in minutes is indicated above the corresponding spectrum. 60 min spectra are deconvoluted using Gaussian–Lorentzian shape components. (C, D) Difference spectra of 60 min minus 1 min. The range is constrained by silicon’s absorption below 1200 cm^–1^ and δ(OH) near 1650 cm^–1^.

Based on NMR spectra presented in Figure S12 in the SI, we consider that at the concentration used for the surface coating (0.1 mg/mL), the main part of the copolymer with unprotected catechol groups, pMPC-*b*-pDOPMA, is fully dissolved and acts as individual molecules, while the main part of the copolymer with acetonide-protected catechol groups, pMPC-*b*-pADOPMA, is in the micellar form. Nevertheless, even in solutions of the copolymer with acetonide-protected catechol groups, an equilibrium between micellized and individual polymer molecules exists, which creates conditions for the adsorption of the copolymers to surfaces. Diblock copolymers pMPC-*b*-pADOPMA and pMPC-*b*-pDOPMA with a DP of the pMPC block 41 and a DP of the pADOPMA (pDOPMA) block 13 were used for adsorption on the gold surface. Adsorption properties of the copolymers were studied *in situ* by employing surface-enhanced infrared absorption spectroscopy (SEIRAS). SEIRAS offers several advantages for the molecular-level investigation of adsorption of the diblock copolymers pMPC-*b*-pADOPMA and pMPC-*b*-pDOPMA on a gold surface. First, SEIRAS is a surface-sensitive technique, and the signal intensity is immensely dependent on the distance from the surface, which allows to infer the structure of supermolecular systems. Second, owing to the metal surface selection rule, SEIRAS provides molecular specificity and enables to work out the orientations of specific molecular groups.^75^ Third, it permits the *in situ* and real-time monitoring of surface processes under the electrochemical stimulus. Figure 5 shows the spectral developments in the 1200–1600 cm^–1^ range associated with adsorption of the copolymers on the gold surface. Given the 3.5:1 molar ratio between the units of MPC and DOPMA in the copolymers, the SEIRAS spectra should predominantly feature MPC bands, specifically, ν_as_(PO_2_) near 1230 cm^–1^ and C–H deformation motion near 1480 cm^–1^. However, that is not the case as the spectra are populated with DOPMA-specific bands near 1255, 1284, 1369, and 1529 cm^–1^, indicating that the interaction between the copolymer and gold primarily ensues through the catechol −OH groups and not phosphorylcholine. In fact, the phosphate spectral mode is vanishingly weak, and thus, the PO_2_ groups are likely to be positioned at a distance from the surface. Time-resolved SEIRAS spectra indicate that the chief adsorption and molecular reorientation occur approximately for the first 10 min, followed by a modest intensity gain and a slight wavenumber shift. A clearer picture emerges from the difference spectrum of 60 min minus 1 min, which shows a concurrent intensity increase of DOPMA-related 1280 cm^–1^ and a decrease of MPC-related C–H deformation at 1481 cm^–1^. Spectral data indicate that at the first adsorption stage (first 1 min), zwitterionic pMPC units are predominantly located near the gold surface; at the second adsorption stage (2–16 min), reorientation of the diblock copolymers proceeds at the surface, resulting in positioning the pDOPMA block directly to the surface. A shift of δ(COH) from 1380 cm^–1^ at 4 min to 1369 cm^–1^ at 60 min suggests establishing a stronger catechol hydrogen bond interaction with the gold surface. Our DFT calculations of DOPMA fragments (Figures S13 and S14) predict the O–H deformation frequency of catechol in vacuum at 1382 cm^–1^, and when hydroxyl groups participate in the hydrogen bond interaction with the Au_3_ cluster (Au···O(H)−), the band downshifts to 1358 cm^–1^. In a previous study, the mode observed at 1363 cm^–1^ of solid-form catechol has shifted to 1356 cm^–1^ due to the adsorption on NaCl crystals, and this shift was attributed to the formation of hydrogen bonds, specifically Na^+^···O(H)–C and Cl^–^···H–O–C.^76^ Alternatively, in the case of the α-Al_2_O_3_ substrate, covalent bonds were established, resulting in a cleavage of the O–H bond and the complete disappearance of the δ(COH) spectral band.^76^ Similarly, dehydrogenation of hydroxyl groups was observed by electron energy-loss spectroscopy for the adsorption of l-DOPA and related compounds on Pt(100) and Pt(111) surfaces.^77^ However, Weinhold et al. demonstrated that the aromatic ring system serves as a primary anchor site for l-DOPA adsorption on the Au(110) surface and the hydroxyl groups do not dehydrogenate.^78^ Given the pronounced strength of the mode at 1369 cm^–1^ in our SEIRAS spectra (Figure 5A), it is evident that in the experiment time frame, catechol–gold interactions can be described as hydrogen bonding-based through the OH groups. The vibrational band associated with the ring stretching mode ν_19_ at 1529 cm^–1^ is also upshifted by 4 cm^–1^ in the surface spectrum; this suggests the possible involvement of a ring moiety in the interaction with the surface.

Figure 5B,D presents the time-resolved spectra related to the surface adsorption of acetonide-protected diblock copolymers pMPC-*b*-pADOPMA. Here, the PO_2_-related spectral mode appears as a strong feature near 1226 cm^–1^ and indicates phosphorylcholine’s close proximity to the gold surface and possible bonding. pADOPMA could be recognized from the 1252, 1491, and 1527 cm^–1^ peaks. These modes remain intense and show minimal time-dependent variability. Notable is a medium intensity feature near 1279 cm^–1^ characteristic of the catechol group without acetonide protection. Its intensity tends to increase with time, evidenced by the positive feature in the difference spectrum. We interpret this mode as evidence of the spontaneous deprotection of some pADOPMA units when they come into contact with a pristine nanostructured gold surface. Weak positive features at 1363 and 1529 cm^–1^ in the difference spectrum are characteristic of pDOPMA and consistent with such an interpretation.

**Figure 6:**
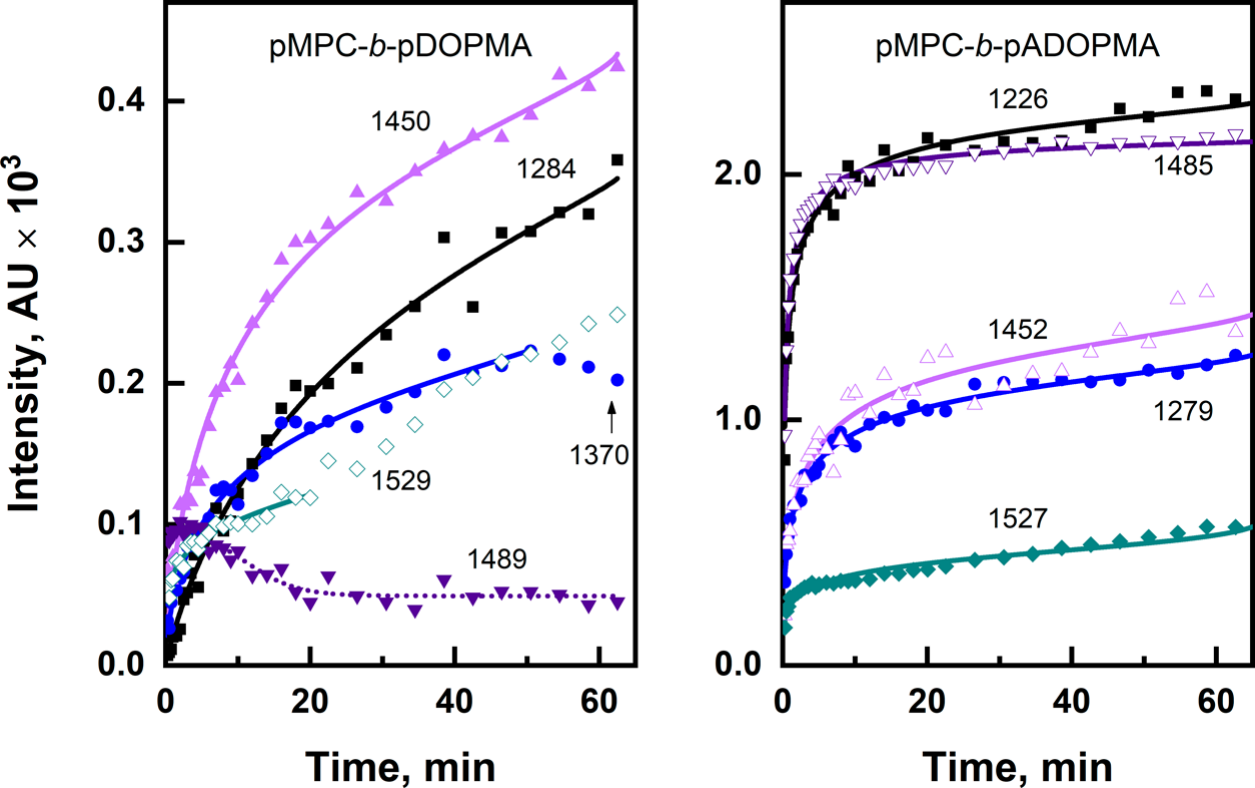
Temporal evolution of selected SEIRAS spectral band intensities of the diblock copolymers pMPC-*b*-pDOPMA and pMPC-*b*-pADOPMA. Experimental data are approximated with the modified Langmuir–Freundlich isotherm (solid lines).

The catechol state in the copolymer, either acetonide-protected or deprotected, strongly affects the adsorption dynamics. Figure 6 shows the spectral modes’ intensity evolutions and the fitted Langmuir–Freundlich (also known as Sips) isotherm. The Langmuir–Freundlich isotherm assumes nonuniform adsorption energy distribution across the surface, which in our case is nanostructured gold.^79^ We also utilize a modified form of this isotherm that is best suited for adsorption from the liquid phase:^79,80^
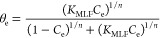
where terms θ_e_ and *K*_MLF_ correspond to the equilibrium surface coverage and the modified Langmuir–Freundlich equilibrium constant, respectively, which correlates with adsorption affinity. θ_e_ is defined as *q*_e_/*q*_m_, where *q*_e_ and *q*_m_ are the amounts of the adsorbed molecules and the saturation capacity, respectively. *C*_e_ is a variable (adsorption time), and *n* is a dimensionless characteristic of adsorption heterogeneity whose value increases with surface heterogeneity. Tabulated values of *K*_MLF_ and *n* are presented in Table S4.

Notably, the intensity evolution is much faster for the acetonide-protected copolymer, and accordingly, the *K*_MLF_ values. The strongest surface affinity of the diblock copolymer pMPC-*b*-pADOPMA is recognized of PO_2_ (1226 cm^–1^) and the protected catechol group (1485 cm^–1^), whose *K*_MLF_ values are 151 and 226, respectively. Other modes exhibited up to 13 times lower constant values and lower spectral intensities in general. The diblock copolymer with unprotected catechol groups, pMPC-*b*-pDOPMA, on the other hand, adsorbed with less acceleration. Although some modes of the unprotected copolymer were impossible to approximate, others exhibited surface affinity factors in single digits of 2.7–5.9. In this case, the C–H deformational band (1450 cm^–1^) was associated with the highest affinity, followed by COH deformation (1370 cm^–1^), and 1284 cm^–1^ mode of catechol (ν(C=C) motion coupled with ν(C–O)). Interestingly, the 1370 cm^–1^ mode’s intensity starts to decline following 50 min of incubation. We presume the cleavage of the CO–H bond and subsequent covalent bonding to gold (Au–O). Furthermore, the pMPC block-associated 1489 cm^–1^ mode, initially dominant, diminishes during the first 20 min. Surprisingly, no modes associated with PO_2_ vibrations, a component of the pMPC block, are detected at any given time during adsorption. Such a complex behavior might illustrate reorientation and competing interactions between pMPC and pDOPMA blocks for the possible adsorption on gold and perhaps unfavored orientation for SEIRAS of some polymer moieties. Lastly, the n parameter is similar between the two copolymers and is above 1, which reports the surface state being heterogeneous.

#### 3.2.3 Desorption of the Copolymers from Gold

**Figure 7:**
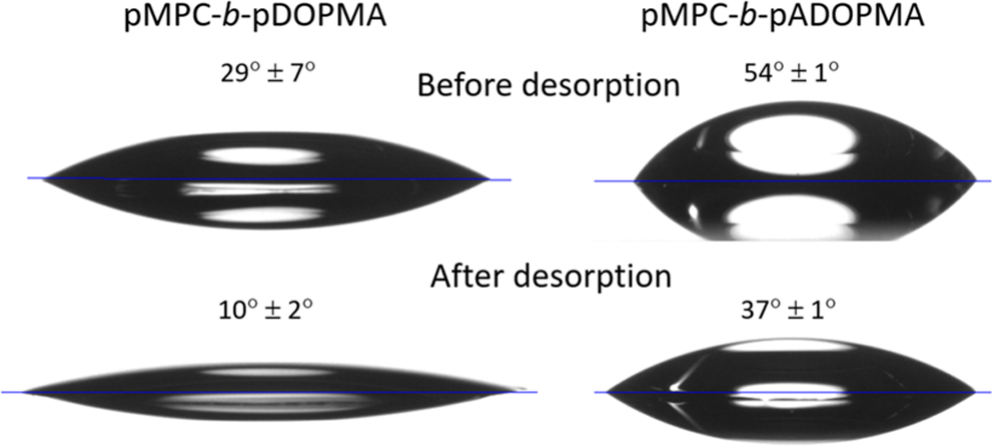
Wetting angles of the diblock copolymers adsorbed on a flat gold film before and after desorption with ultrasound in methanol for 15 min.

**Figure 8:**
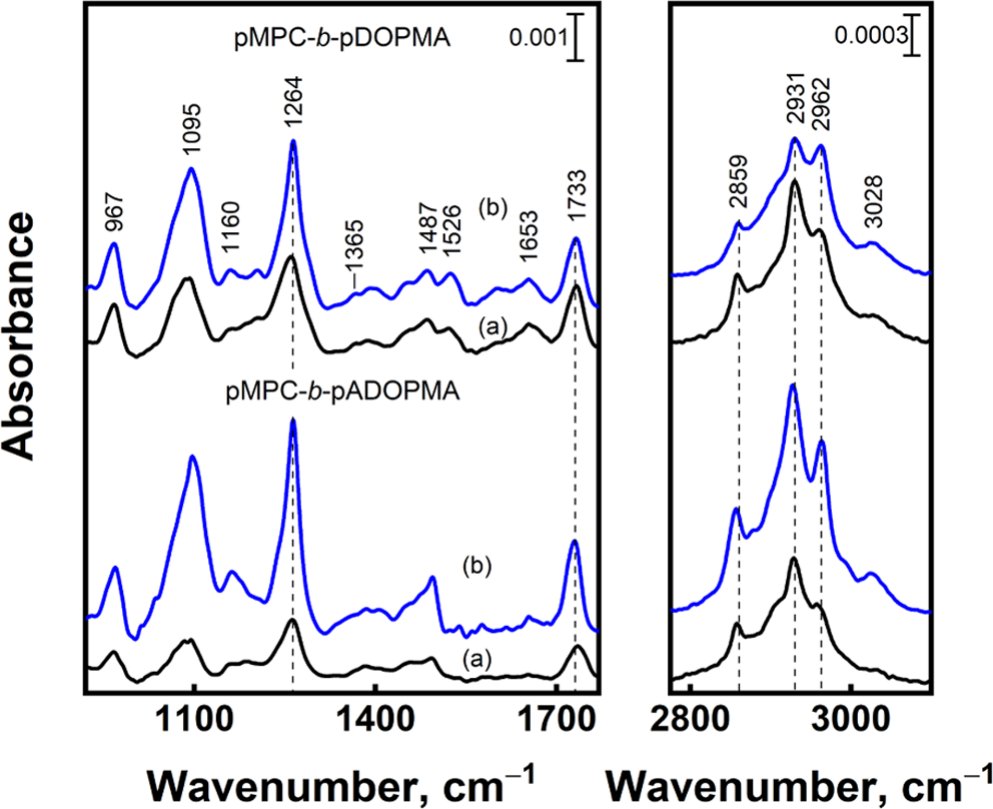
RAIRS spectra of the gold surface with adsorbed diblock copolymers (b) before and (a) after ultrasonication in methanol for 15 min.

Desorption of the diblock copolymers pMPC-*b*-pDOPMA and pMPC-*b*-pADOPMA from a 150 nm magnetron sputtered gold film was performed in an ultrasonic bath in methanol for 15 min, followed by thorough rinsing. Such a gold surface may be considered reasonably smooth with a roughness factor of approximately 1.3 and dominated by a (111) facet.^81^ Wetting behavior and reflection-absorption infrared spectroscopy (RAIRS) data were recorded before and after desorption (Figures 7 and 8). Prior to desorption, the film of the diblock copolymer pMPC-*b*-pDOPMA already exhibited a more strongly pronounced hydrophilicity with a contact angle of sessile drop equal to 29°, compared to 54° of the acetonide-protected copolymer pMPC-*b*-pADOPMA. These findings are consistent with the SEIRAS results, which demonstrated that hydrophilic phosphorylcholine head groups in the copolymers pMPC-*b*-pDOPMA were set farther from the gold surface, likely extending into the bulk. Following ultrasonic treatment, the wetting angles decreased by nearly 20° for both copolymers, indicating an additional increase in hydrophilicity. Several processes may explain the observed changes: first, the mechanical removal of polymer adlayers and weakly adsorbed polymer molecules and second, the reorientation of the adsorbed polymer chains such that hydrophilic phosphorylcholine head groups become more exposed to the ambient environment. In order to deepen our understanding of molecular-level changes, we conducted RAIRS analysis. Both aspects, the removal and the reorientation of molecules, may contribute to the RAIRS spectra because the intensity of the RAIRS signal depends on the amount of material and the surface selection rule (dipole orientation with respect to the surface determines the spectral mode intensity). However, unlike tightly packed alkanethiol monolayers,^82^ polymer chains are much less spatially constrained holding no particular arrangement, which leads to the reduction in spectral sensitivity to polymer chain orientation. Thus, in this case, RAIRS foremost predicts changes in the amount of adsorbed material. We found 66% reduction in the area under the curve within the 750–1800 cm^–1^ range for the acetonide-protected copolymer and only a 29% reduction for the copolymer with unprotected catechol groups. Thus, less material was removed from the surface for the copolymer with unprotected catechol groups as it has a higher extent of surface-active sites (the catechol group and the phosphorylcholine head group).

Based on the surface selection rule, the intensity of the ν(=C–H) mode at 3028 cm^–1^ is sensitive to benzene ring orientation. The integral intensity decrease (with respect to the integral intensity of the 2750–3200 cm^–1^ range) was only 15% for the copolymer pMPC-*b*-pDOPMA and 45% for the copolymer pMPC-*b*-pADOPMA. This observation attests the maintained orientation of the unprotected catechol on a gold surface and the more flexible orientation of the protected catechol. The relative intensity of ν_s_(CH_2_) and ν_as_(CH_2_) bands at 2859 and 2931 cm^–1^, respectively, increases after the ultrasonication procedure, while the relative intensity of ν_as_(CH_3_) and ring ν(=C–H) modes at 2962 and 3028 cm^–1^, respectively, decreases after sonication, indicating the preservation of a more flat orientation of the ring group at the surface for the adsorbed copolymer pMPC-*b*-pDOPMA. In the case of the copolymer with acetonide-protected catechol groups pMPC-*b*-pADOPMA, all of the bands in the high-frequency spectral region (2800–3100 cm^–1^) considerably decrease in intensity, indicating different adsorption states of the copolymer.

**Figure 9:**
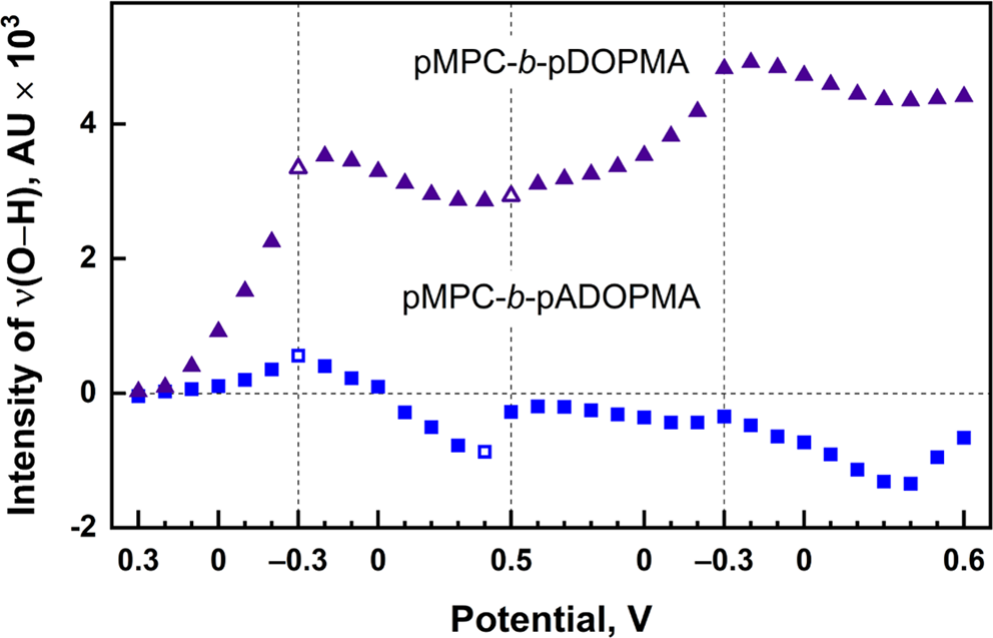
Dependence of the ν(O–H) integral intensity of water on electric potential with respect to the Ag/AgCl reference electrode for the gold surface with adsorbed diblock copolymers pMPC-*b*-pDOPMA and pMPC-*b*-pADOPMA. Potential limits are marked by dashed vertical lines. Open symbols indicate the water spectra presented in Figure S15.

Wetting properties of the diblock copolymer pMPC-*b*-pDOPMA adsorbed on nanostructured gold and its acetonide-protected counterpart were studied by using SEIRAS under an external electric potential. Figure 9 shows the dependency of the spectral intensity of the O–H stretching vibrational mode of the polymer-proximal water in the two cycles of potential cycling. Over the whole potential window, the copolymer with unprotected catechol groups shows the capacity to attract water molecules to an extent far greater compared to the copolymer pMPC-*b*-pADOPMA. Such an observation aligns with temporal SEIRAS evolution and contact angle measurements that suggested the exposure of phosphorylcholine groups to the environment. Water near the copolymer pMPC-*b*-pADOPMA, on the other hand, shows little sensitivity to the potential and is distanced from the surface at most of the potentials tested. Despite noticeable alterations in the spectral intensity, the potential seems to have a minimal impact on the composition of interfacial water (Figure S15 in the SI). Specifically, for pMPC-*b*-pADOPMA, a frequency upshift of 40–50 cm^–1^ was observed for water band components at −0.4 V, suggesting the potential-induced withdrawal of water molecules engaged in weaker hydrogen bonding.^83,84^ In conclusion, SEIRAS and contact angle measurements demonstrate that under varying external electric potentials, the copolymer with unprotected catechol groups pMPC-*b*-pDOPMA significantly outperforms its acetonide-protected counterpart in attracting water molecules, likely due to the exposed phosphorylcholine groups.

**Figure 10:**
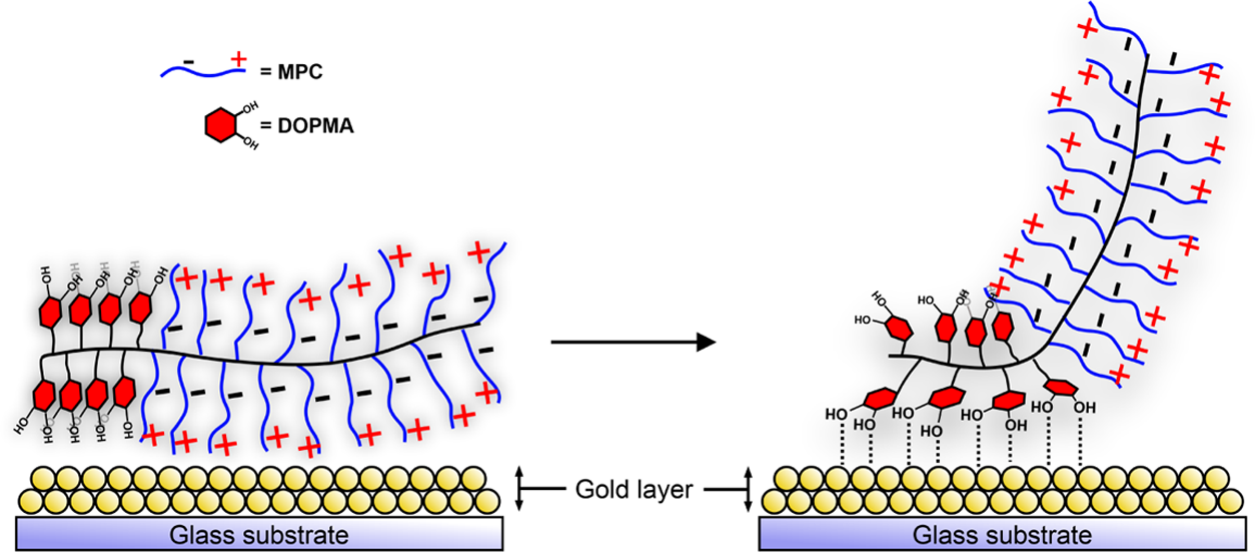
Schematic illustration of the time evolution of the structure of the block copolymers pMPC-*b*-pDOPMA on the gold surface during the adsorption process.

In conclusion, the present study demonstrates that the adsorption of the diblock copolymers pMPC-*b*-pDOPMA on the gold surface is a prolonged process. At the first adsorption stage, zwitterionic phosphorylcholine groups are predominantly located near the gold surface, but later the reorientation of the diblock copolymers proceeds at the surface resulting in positioning the pDOPMA block directly to the surface. These data indirectly indicate that in adsorbed layers of the diblock copolymers pMPC-*b*-pDOPMA, the block of pMPC is likely extended into the bulk, forming a highly hydrated layer (Figure 10). Such a vision is in accord with previous studies showing excellent swelling of surface-anchored pMPC chains, which was demonstrated by the increased root-mean-square roughness of the coated surface upon prewetting with pure water immersion.^57^ It was confirmed by atomic force microscopy (AFM) examination that surface hydrophilicity was enhanced by the orientation of zwitterionic units of the pMPC block toward the aqueous phase.^57^

## 4 Conclusions

Amphiphilic diblock copolymers containing a block of 2-methacryloyloxyethyl phosphorylcholine (MPC) with unique properties to prevent nonspecific protein adsorption and enhance lubrication in aqueous media and a block of dopamine methacrylamide (DOPMA) distinguished by excellent adhesion performance were synthesized by RAFT polymerization. The DOPMA monomer with an acetonide-protected catechol group (ADOPMA) was used for the synthesis, allowing the prevention of undesirable side reactions during polymerization and preserving the synthesized copolymers from oxidation during storage. The diblock copolymers synthesized starting from the pMPC block contained a short pDOPMA block, with a DP of not more than 13, which was predetermined by insufficient solubilization of the growing pADOPMA chains. The diblock structure of the copolymers was substantiated by ^1^H NMR and ^13^C NMR spectra as well as by DLS particle size distribution curves of the copolymers with protected and unprotected catechol groups in various solvents.

The adsorption behavior of the diblock copolymers pMPC-*b*-pADOPMA and pMPC-*b*-pDOPMA on gold surfaces was probed using infrared absorption spectroscopic techniques, specifically, ATR-FTIR spectroscopy, surface-enhanced infrared absorption spectroscopy (SEIRAS), and reflection–absorption infrared spectroscopy (RAIRS). It was determined that the PO_2_-related vibrational band at 1226 cm^–1^ and catechol-related modes in the vicinity of 1369 and 1529 cm^–1^ were instrumental in elucidating the adsorption dynamics between polymeric blocks carrying phosphorylcholine and dopamine fragments. The diblock copolymer with acetonide-protected catechol groups pMPC-*b*-pADOPMA demonstrated a physisorption behavior marked by rapid and intense adsorption and a significant degree of polymer desorption using ultrasound. In contrast, the diblock copolymer with unprotected catechol groups pMPC-*b*-pDOPMA exhibited chemical adsorption characteristics, evidenced by slower adsorption dynamics, a stronger interaction with the gold surface during desorption with ultrasound, and considerable spectral shifts and intensity changes in vibrational modes associated with catechol groups. Spectral data indicate that at the first adsorption stage, zwitterionic phosphorylcholine groups are predominantly located near the gold surface, but later the reorientation of the diblock copolymers proceeds at the surface, resulting in positioning the pDOPMA block directly to the surface. At the same, as it was shown by SEIRAS measurements under varying electric potentials and contact angle analysis, phosphorylcholine groups were abundantly exposed, which was evidenced by the superior hydrophilicity of the decorated surface and water molecule attraction. These data indirectly indicate that in adsorbed layers of the diblock copolymers pMPC-*b*-pDOPMA, the block of pMPC is likely extended into the bulk, forming a highly hydrated layer.

## Data Availability

Data will be made available on request.

## Supporting Information Available

Materials, synthesis details, NMR spectra, calculations, UV–vis spectra, calibration graph, theoretical modeling of DOPMA adsorption onto the Au_3_ cluster, and calculated infrared spectra (PDF)
