# Supplementary material for: Diblock Copolymers of Methacryloyloxyethyl Phosphorylcholine and Dopamine Methacrylamide: Synthesis and Real-Time Adsorption Dynamics by SEIRAS and RAIRS
Source: Langmuir. 2024 Mar 8;40(11):5945–58. doi: 10.1021/acs.langmuir.3c03925 (PMC10956495; doi:10.1021/acs.langmuir.3c03925)
Supplement: Supplementary file 1 — la3c03925_si_001.pdf [file la3c03925_si_001.pdf]

Supporting Information

Diblock copolymers of methacryloyloxyethyl phosphorylcholine and dopamine methacrylamide: synthesis and real-time adsorption dynamics by SEIRAS and RAIRS

Marijus Jurkūnas[1*‡], Martynas Talaikis[2‡], Vaidas Klimkevičius[1], Vaidas Pudžaitis[2], Gediminas Niaura[2], Ričardas Makuška[1]

[1]Institute of Chemistry, Vilnius University, Naugarduko str. 24, 03225 Vilnius, Lithuania

[2]Department of Organic Chemistry, Center for Physical Sciences and Technology (FTMC), Sauletekio Ave. 3, 10257 Vilnius, Lithuania

*marijus.jurkunas@chgf.vu.lt

‡ M.J. and M.T. contributed equally to this work.

Table of contents

1. Materials 2
2. Synthesis 2
   2.1. Synthesis of the RAFT chain transfer agent (CTA) 2
   2.2. Synthesis of acetonide protected dopamine methacrylamide (ADOPMA) 4
   2.3. Removal of acetonide protective group 4
3. Calculations 7
   3.1. Calculation of copolymer composition from UV-Vis spectra 7
   3.2. Calculation of copolymer composition from 1H NMR spectra 9
4. Approval of copolymer structure using NMR spectra 9
5. Theoretical modelling of DOPMA adsorption onto Au_3 cluster 10
6. Literature 12

1. Materials

Dopamine hydrochloride, 2,2-dimethoxypropane (98%, Aldrich), *p*-toluenesulfonic acid monohydrate (p-TsOH, 98%, Aldrich), sodium tetraborate decahydrate ($Na_2B4O_7 \cdot 10H_2O$), methacrylic anhydride (94%, Aldrich), trifluoroacetic acid (TFA), sodium carbonate (Na_2CO_3), magnesium sulfate ($MgSO_4$) were of the highest grade, purchased from Sigma-Aldrich and used without further purification. Hexane (Aldrich), dichloromethane (Acros), ethyl acetate (Fischer Scientific), diethyl ether (Aldrich), methanol (Aldrich), anhydrous toluene (Aldrich), anhydrous *N,N*-dimethylformamide (DMF, Aldrich, 99.9%), sodium hydride (NaH, 60% in mineral oil, Aldrich), 1-butanethiol (BuSH, 99%, Aldrich), carbon disulfide (CS_2, 99%, Aldrich), sodium iodide (≥ 99.5%, Aldrich), sodium thiosulfate (99%, Aldrich) were used as received.

2. Synthesis

2.1. Synthesis of the RAFT chain transfer agent (CTA)

4-(((Butylthio)carbonothioyl)thio)-4-cyanopentanoic acid (BCPA) was synthesized according to the general procedure described previously with few adjustments. To a cold (0 °C) solution of 1-butanthiol (2.89 g, 32.0 mmol) in anhydrous diethyl ether (70 mL) 1.45 g NaH (60% in mineral oil, 36.3 mmol) was continuously added. After 30 min of stirring, 12.18 g of CS_2 (160 mmol) were added dropwise at 0 °C. The cloudy yellow reaction mixture containing the formed sodium butyl carbonotrithionate was allowed to warm to room temperature. Later on, the mixture was 30 min purged with N_2 gas, then 5.08 g of iodine (20 mmol) were added in one portion, and the reaction mixture was stirred for 1 hour at room temperature. The formed precipitate of insoluble NaI in diethyl ether were removed by filtration, and the filtrate was washed several times using 1 M $Na_2S_2O_3$ aqueous solution to remove unreacted iodine. The combined organic layers were dried with $MgSO_4$, and the solvent was removed under reduced pressure giving 6.0 g of yellow viscous oil of bis-(butylsulphanylthiocarbonyl) disulfide. 4,4-Azobis(4-cyanovaleric acid) (ACVA) (13.44 g, 48.0 mmol) was added into three-neck round-bottom flask containing 6.0 g of bis(butyltrithiocarbonate) dissolved in 150 mL of EtOAc, and the solution was stirred overnight under reflux in N_2 atmosphere. The solution was washed with water (3 × 100 mL) to remove unreacted ACVA, and concentrated using rotary evaporator. The product was purified using flash column chromatography (eluent hexanes:EtOAc:AcOH = 4:1:0.01 (v/v), R_f 0.2), and the solvent was removed resulting in yellow solid 4-(((butylthio)carbonothioyl)thio)-4-cyanopentanoic acid (BCPA). Overall yield 8.29 g (89%). Analytical data of BCPA: m.p. 41-45 °C; ^{1}H NMR (400 MHz, $CDCl_3$) ppm: 0.96 (t, *J* = 7.4 Hz, 3H), 1.45 (m, 2H); 1.71 (m, 2H), 1.91 (s, 3H), 2.36–2.60 (m, 2H), 2.71 (t, *J* = 7.8 Hz 2H), 3.36 (t, *J* = 7.5 Hz, 2H); ^{13}C NMR (100 MHz, $CDCl_3$) ppm: 13.59, 22.10, 24.86, 29.49, 29.70, 33.48, 36.78, 46.20, 118.90, 177.12, 216.79.

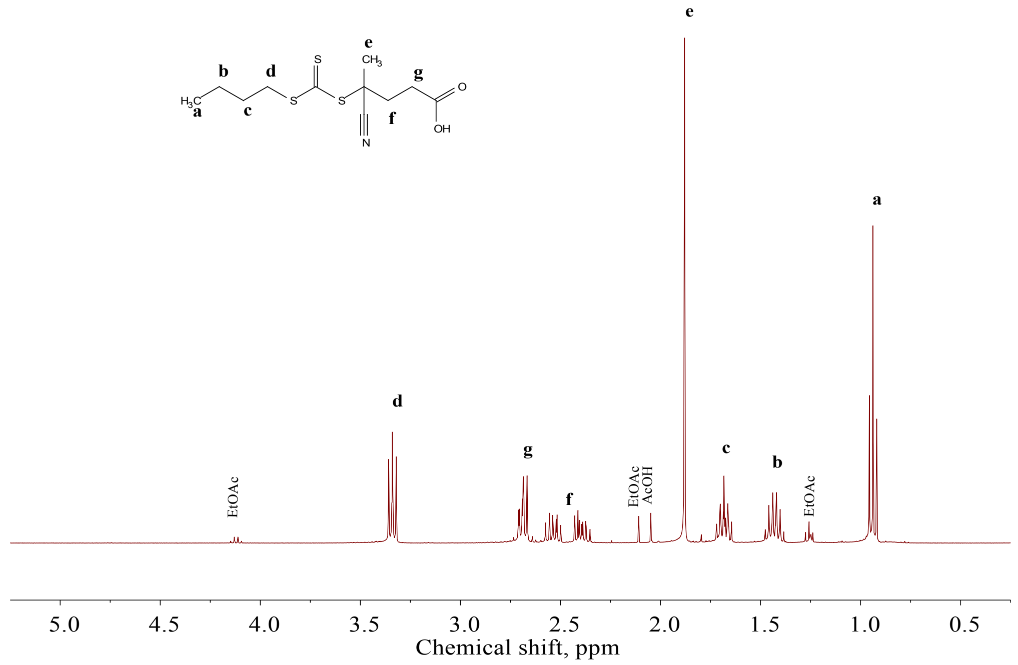

Figure S1. ^{1}H NMR spectrum of 4-(((butylthio)carbonothioyl)thio)-4-cyanopentanoic acid (BCPA) in $CDCl_3$ at 22 °C.

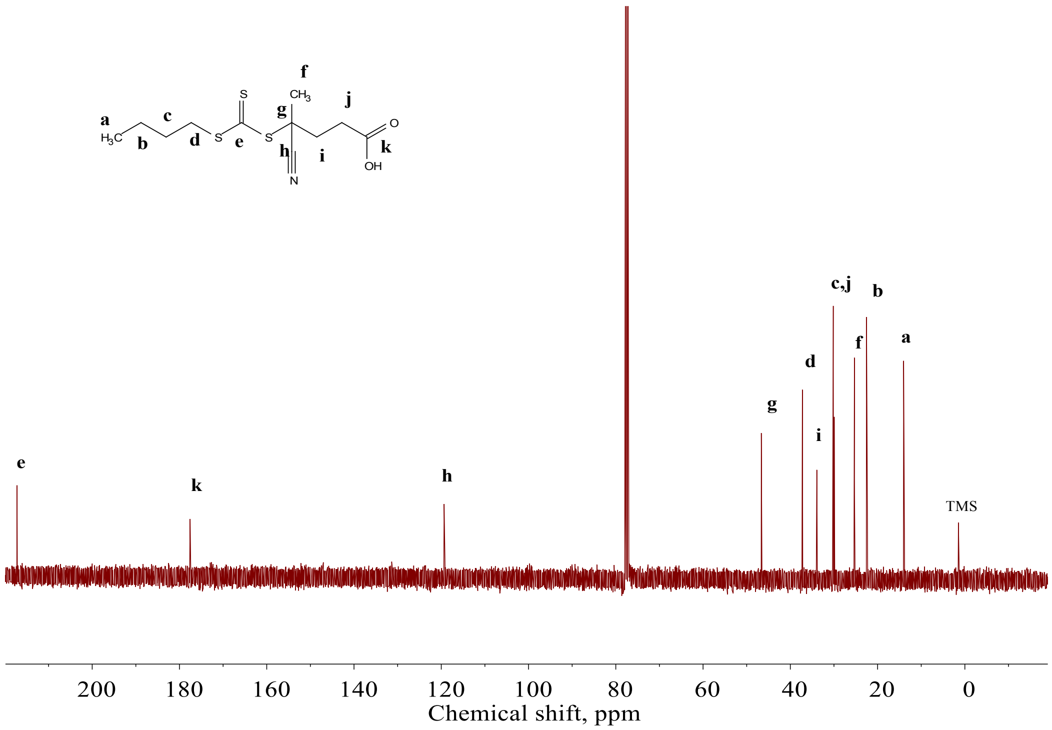

Figure S2. ^{13}C NMR spectrum of 4-(((butylthio)carbonothioyl)thio)-4-cyanopentanoic acid (BCPA) in $CDCl_3$ at 22 °C.

2.2. Synthesis of acetonide protected dopamine methacrylamide (ADOPMA)

DOPMA was synthesized according to the procedure published before [1]. 20 g (52.44 mmol) of sodium borate decahydrate ($Na_2B_4O_7 \cdot 10H_2O$) and 8 g (95.23 mmol) of sodium bicarbonate ($NaHCO_3$) were dissolved in 200 mL of deionized water, the solution was bubbled with nitrogen gas for 30 min, and then 10 g (52.8 mmol) of dopamine hydrochloride were poured to the aqueous mixture of sodium borate/sodium bicarbonate under nitrogen flow. After that, 9.5 mL (58.1 mmol) of methacrylic anhydride dissolved in 50 mL THF was added dropwise under constant stirring. During the reaction between 3,4-dihydroxyphenethylamine hydrochloride and methacrylic anhydride, pH of the solution was kept moderately above 8 by adding 1 M aqueous NaOH solution. After stirring for 24 h at room temperature under nitrogen, the aqueous reaction mixture was washed twice with 100 mL of ethyl acetate to remove residual methacrylic anhydride and then acidified to pH < 2 with concentrated HCl. After extraction (three times with 100 mL of ethyl acetate), the combined brown organic phase was dried over $MgSO_4$. The solvent was removed using rotary evaporator, and the product was crystallized and recrystallized from hexane–ethyl acetate mixture (7:3, v:v). The obtained slightly greyish powder was dried in a vacuum-oven overnight at room temperature, yield 95%. ^{1}H NMR (250 MHz, DMSO-d_6, δ(ppm)): 8.12 (t, 1H, -NH-C=O), 6.56-6.74 (m, 3H, Ph), 6.21 (dd, 1H, -C$\underline{H}$=CH2), 6.05 (dd, 1H, C$\underline{H_2}$=CH-), 5.56 (dd, 1H, C$\underline{H_2}$=CH-), 3.30 (q, 2H, -CH2-C$\underline{H_2}$-NH-), 2.63 (t, 2H, -C$\underline{H_2}$-CH_2-NH-), 1.61 (s, 6H, $(CH_3)_2$-C-). ^{13}C NMR (400 MHz, DMSO-d_6, δ(ppm)): 164.6 (1C, -NH-C=O), 146.8 (1C, $\underline{Ph}$-O(C(Me)$_2$)), 145.2 (1C, $\underline{Ph}$-O(C(Me)$_2$)), 132.6 (1C, -$\underline{C}$H=CH_2), 131.8 (1C, $\underline{Ph}$-CH_2-), 124.8 (1C, -CH=$\underline{C}H_2$), 120.9 (1C, Ph), 117.6 (-$\underline{C}(CH_3)_2$), 108.7 (1C, Ph), 107.6 (1C, Ph), 40.4 (1C, -CH_2-NH-), 34.7 (1C, - 80 $\underline{C}H_2$-CH_2-), 25.5 (1C, ($\underline{C}H_3)_2$-C-).

2.3. Removal of acetonide protective group

Removal of the acetonide protective groups from catechol moieties present in pADOMPA or diblock copolymers pMPC-*b*-pADOPMA was performed using TFA [1]. In a round-bottomed flask, 0.2 g of the copolymer was dissolved in 7.4 mL of dichloromethane and 0.1 mL of deionized water, and the mixture was bubbled with nitrogen gas for 20 min. Then the mixture was cooled in an ice bath, and 2.5 mL of TFA was added under vigorous stirring. After 30 min the solution was left stirring for 60 min at room temperature. The copolymer was purified by dialysis through 3.5 kDa MWCO regenerated cellulose membrane against water, and the solid white powder was obtained by freeze-drying.

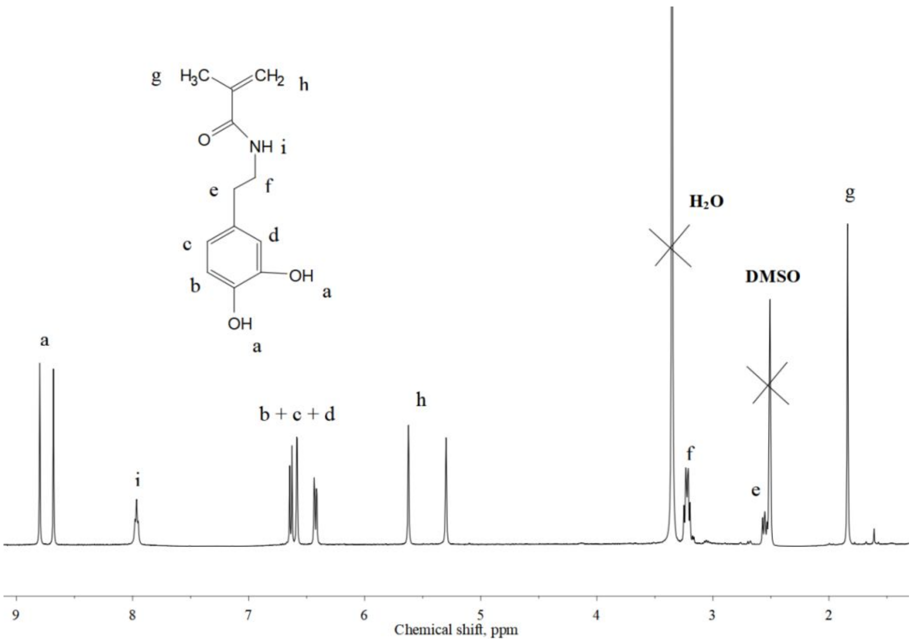

Figure S3. ^{1}H NMR spectrum of DOPMA in DMSO-d_6 at 22 ^{o}C

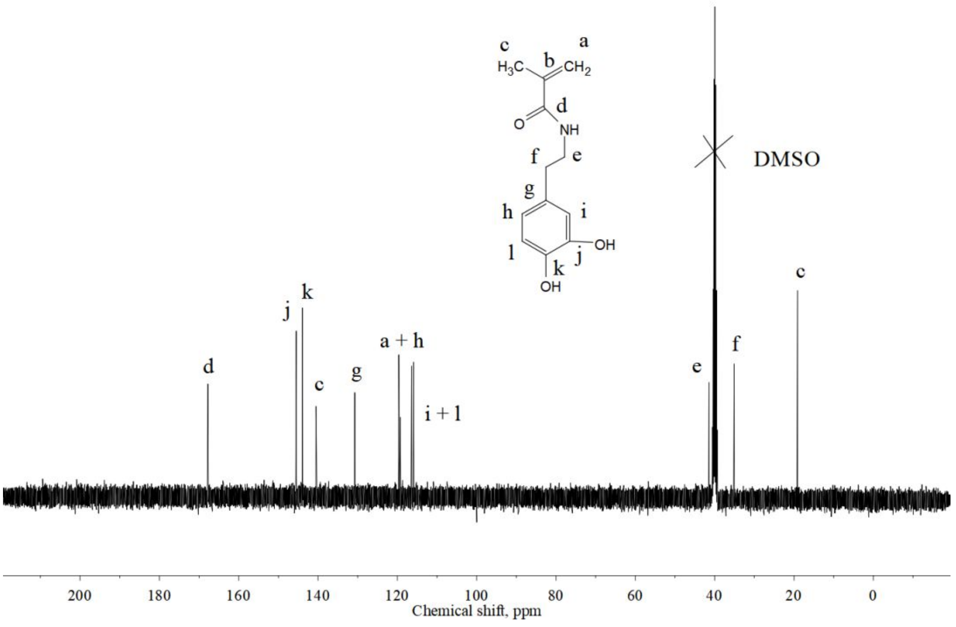

Figure S4. ^{13}C NMR spectrum of DOPMA in DMSO-d_6 at 22 ^{o}C.

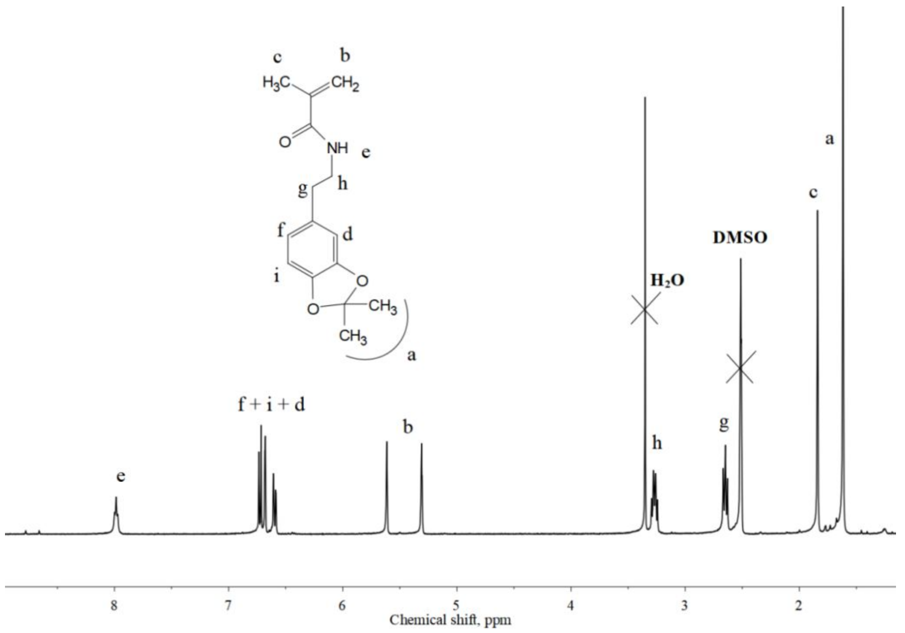

Figure S5. ^{1}H NMR spectrum of ADOPMA in DMSO-d_6 at 22 ^{o}C

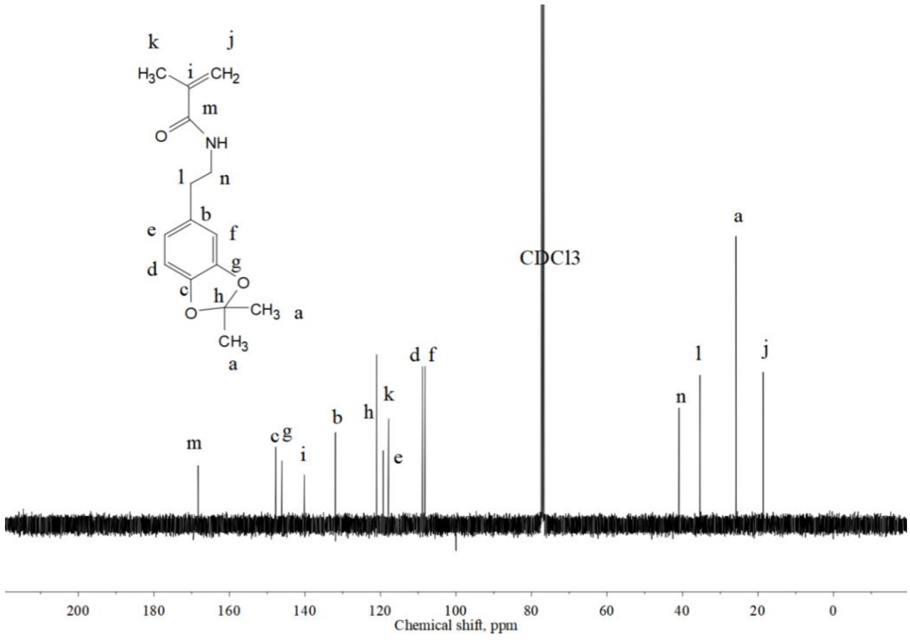

Figure S6. ^{13}C NMR spectrum of ADOPMA in $CDCl_3$ at 22 ^{o}C.

3. Calculations

3.1. Calculation of copolymer composition from UV-Vis spectra

A series of pADOPMA absorption spectra in methanol showed typical benzene ring absorption peak at 289 nm (Figure S7). Solutions of pMPC showed wide but weaker than pADOPMA absorption peak ranging from 270 nm to 350 nm (maximum at 310 nm). Intensity of pADOPMA peak was corrected by subtracting intensity of pMPC absorption at 289 nm. The calibration graph absorption of ADOPMA versus concentration was used for determination of the amount of monomeric units carrying benzene ring in the copolymers pMPC-*b*-pADOPMA (Figure S9). Results of determination are summarized in **Table S1.**

Table S1. Copolymer composition calculation from UV-Vis data

Copolymer	Concentration, mg/mL	Absorbance at 289 nm, a.u.	Corrected absorbance, a.u.	ADOPMA in copolymer, mg	Copolymer composition, ADOPMA mol%
pMPC-*b*-pADOPMA	0.25	0.64	0.57	0.037	16.4
	0.5	1.25	1.07	0.074	16.4
	1	2.44	2.05	0.147	16.3
pMPC	0.25	0.08			
	0.5	0.18			
	1	0.39			

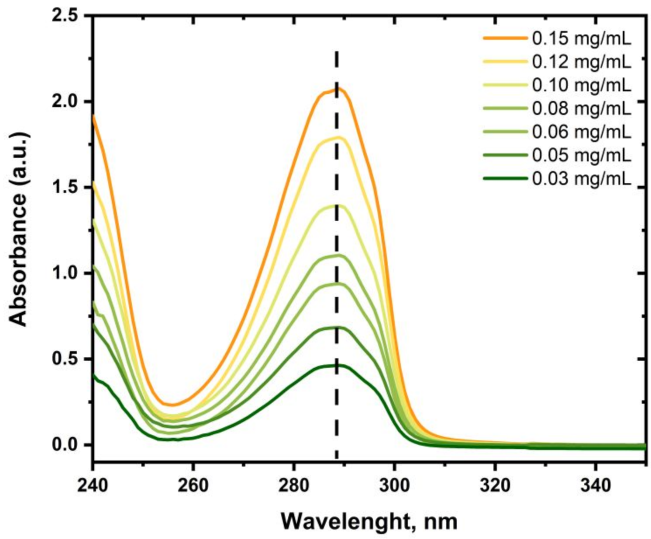

Figure S7. UV-VIS spectra of pADOPMA in MeOH. The data of polymer absorbance at 289 nm is used for calibration graph.

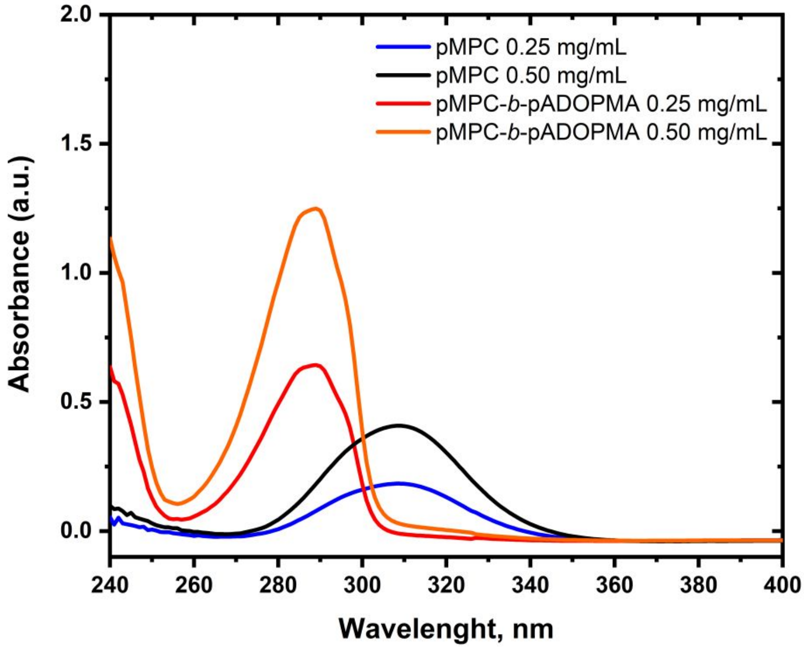

Figure S8. UV-Vis spectra of pMPC and pMPC-*b*-pADOPMA in MeOH.

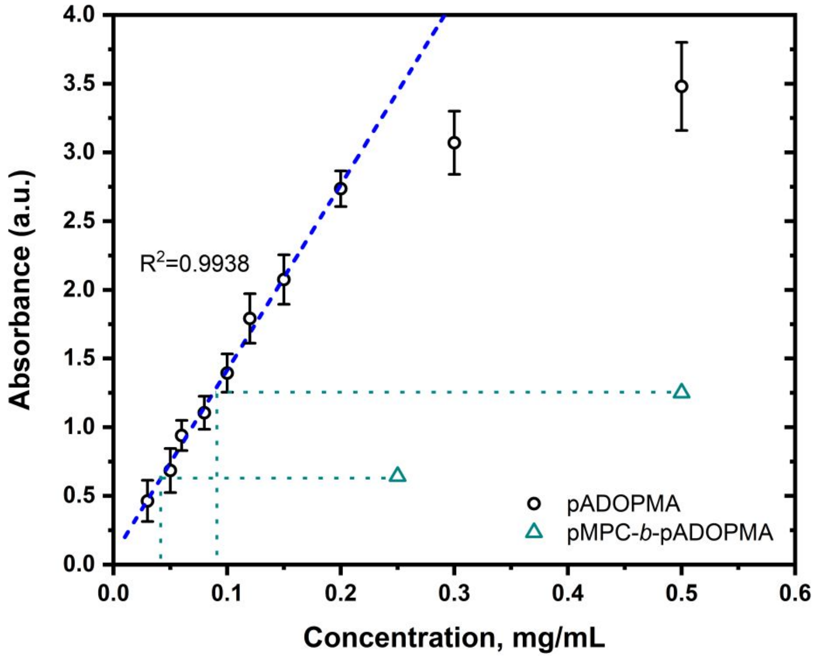

Figure S9. Calibration graph absorbance versus concentration of ADOPMA; and absorbance of pMPC-*b*-pADOPMAat 289 nm.

3.2. Calculation of copolymer composition from 1H NMR spectra

Table S2. Composition of the diblock copolymers calculated from ^{1}H NMR and UV-VIS spectra.

Diblock polymer	Monomeric unit	Attributed functional group	Chemical shift, ppm	Integral area	Integral area per proton	Molar fraction by NMR, %	Molar fraction by UV-VIS, %	DP in polymeric block
pMPC-*b*-pADOPMA	MPC	-N(C$\underline{H}_3$)$_3$	3.27	49.98	5.554	83.5	83.6 ± 0.1	34*
		-PO$_4$-CH$_2$-C$\underline{H}_2$-N-	3.72	10.14	5.068			
		-C$\underline{H}_2$-C$\underline{H}_2$-PO$_4$-C$\underline{H}_2$-	4.3-4.0	29.69	4.948			
	ADOPMA	-C(C$\underline{H}_3$)$_2$	1.56	6.000	1.000	16.5	16.4 ± 0.1	7
		-NH-CH$_2$-C$\underline{H}_2$-	2.61	2.040	1.021			
		Benzene ring (3H)	6.50	2.659	0.886			

* DP calculated from SEC analysis.

4. Approval of copolymer structure using NMR spectra

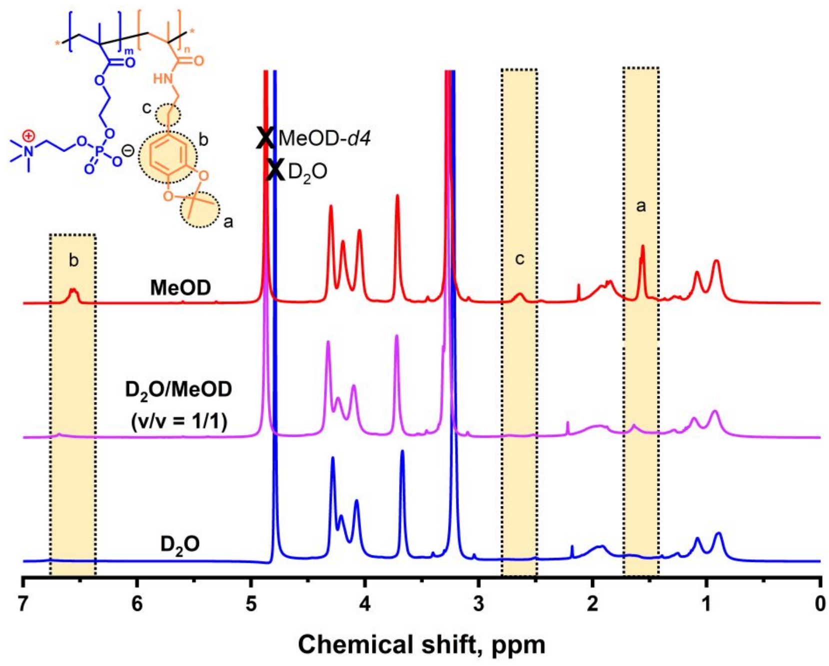

Figure S10. ^{1}H NMR spectra of the diblock copolymer pMPC-*b*-pADOPMA in D_2O, MeOD-*d4*/D_2O mixture and MeOD-*d4*. The appearance of chemical shifts characteristic for pADOPMA block (marked in yellow) in MeOD-containing solutions proved diblock structure of the copolymer.

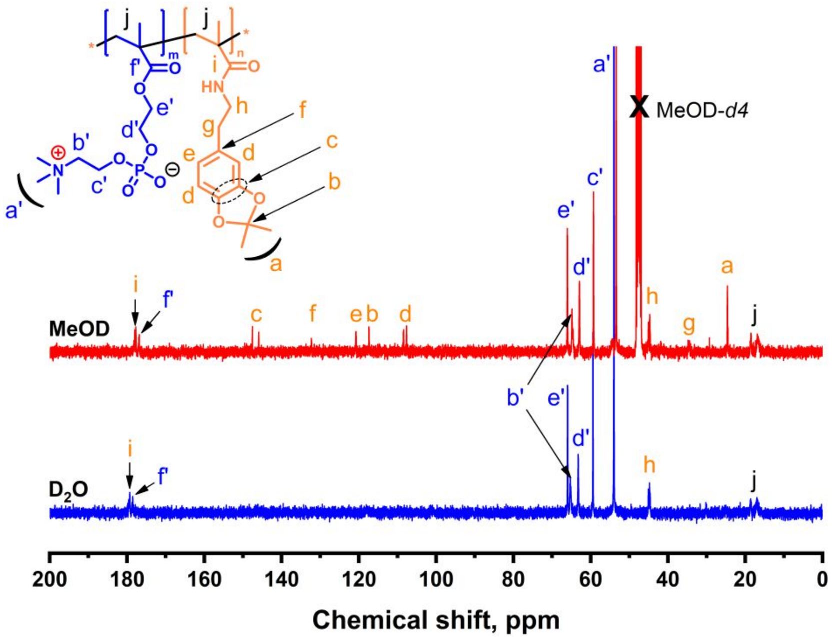

Figure S11. ^{13}C NMR spectra of the diblock copolymer pMPC-*b*-pADOPMA in D_2O and MeOD-*d4*.

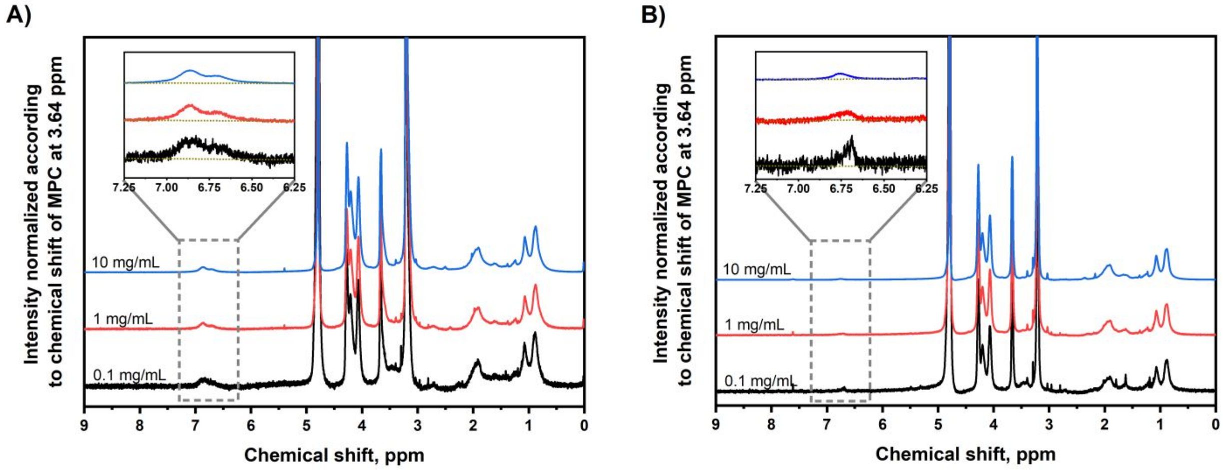

Figure S12. ^{1}H NMR spectra of the diblock copolymers with unprotected catechol groups pMPC-*b*-pDOPMA (A) and with acetonide-protected catechol groups pMPC-*b*-pADOPMA (B) in D_2O at various concentrations of the copolymers.

Table S3. DOPMA (ADOPMA) content in the copolymers pMPC-*b*-pDOPMA and pMPC-*b*-pADOPMA determined from ^{1}H NMR spectra of the copolymers in D_2O solutions of various concentrations.

Copolymer	Deuterated solvent	Concentration, mg/mL	DOPMA (ADOPMA) content, mol%
pMPC-*b*-pDOPMA	MeOD-*d*$_4$	10	24.1
	D_2O	10	12.6
		1	13.7
		0.1	20.7
pMPC-*b*-pADOPMA	MeOD-*d*$_4$	10	24.1
	D_2O	10	3.1
		1	5.1
		0.1	6.4

5. Theoretical modelling of DOPMA adsorption onto Au_3 cluster

Theoretical modelling of DOPMA fragment and DOPMA fragment with Au_3 cluster was performed using Gaussian 09 for Windows [2]. Geometry optimization and vibrational frequency calculations were performed using the density functional theory (DFT) method and the B3LYP functional. Calculations were accomplished using the 6-311++G(2d,p) basis set for C, H, and O atoms and LANL2DZ with ECP for gold atoms. The cluster model built from 3 gold atoms represents the metal surface. Calculated vibrational frequencies and intensities were scaled according to the method described elsewhere [3].

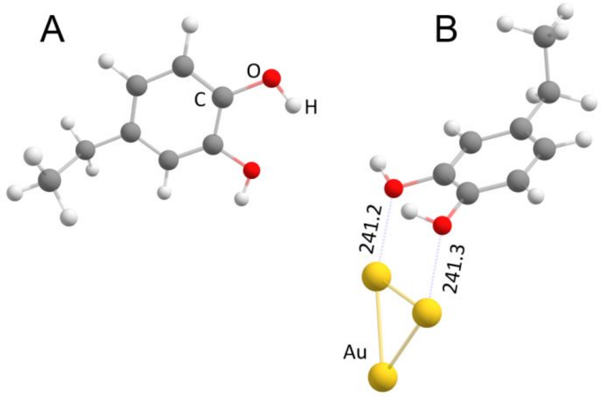

Figure S13. (A) Optimized geometries of DOPMA fragment (4-ethylbenzene-1,2-diol) and (B) the Au_3-DOPMA fragment complex. The interatomic Au···O distances are indicated in picometers.

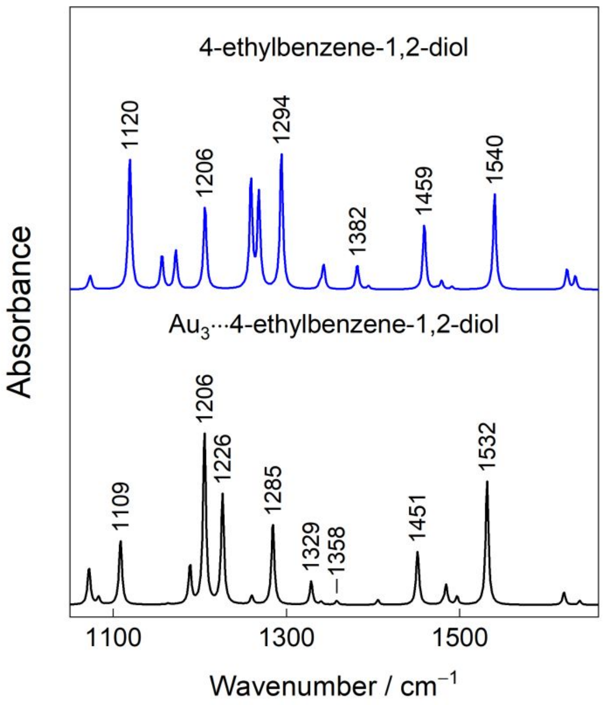

Figure S14. Theoretical spectra for the DOPMA fragment (4-ethylbenzene-1,2-diol) and its complexes with Au_3 cluster.

Table S4. K_{LF} and n Parameters Derived from Spectral Band Intensity Evolution Approximated with Modified Langmuir-Freundlich Isotherm

pMPC-*b*-pDOPMA		
ν / cm^{-1}	K_{MLF}	n
1450	5.7 ± 0.5	1.3 ± 0.1
1284	2.7 ± 0.2	1.1 ± 0.1
1370	5.9 ± 1.1	1.5 ± 0.1

pMPC-*b*-pADOPMA		
ν / cm^{-1}	K_{MLF}	n
1226	151 ± 12	1.8 ± 0.1

1485	226 ± 10	1.3 ± 0.0
1452	27 ± 4.5	1.8 ± 0.2
1279	39 ± 2.5	1.9 ± 0.1
1527	17 ± 3.5	2.9 ± 0.2

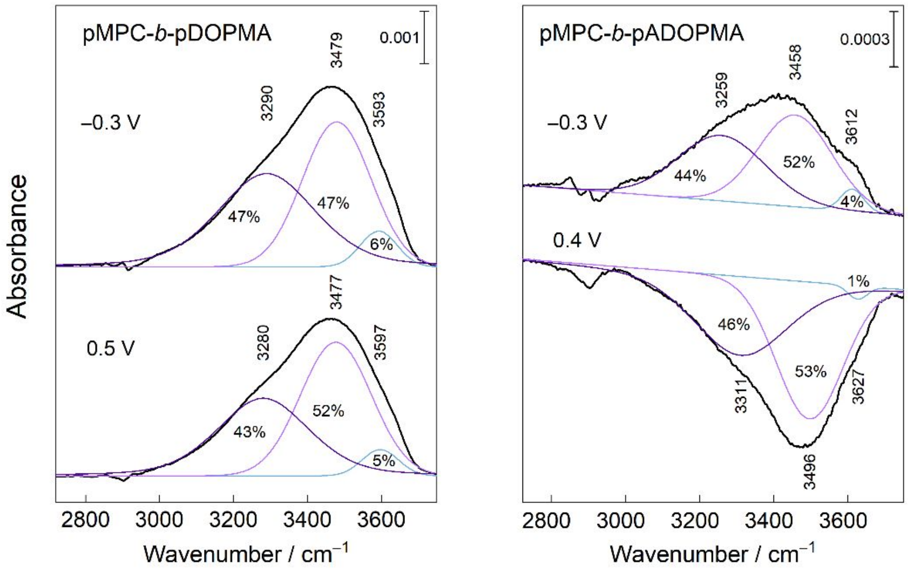

Figure S15. SEIRAS spectra of interfacial water at selected electric potential during the first potential cycle. The deconvolution was carried out using Gaussian-Lorentzian shape components.

6. Literature

[1] M. Steponaviciute, V. Klimkevicius, R. Makuska, Synthesis and stability against oxidation of random brush copolymers carrying PEO side chains and catechol moieties, Mater. Today Commun. 25 (2020) 101262. https://doi.org/https://doi.org/10.1016/j.mtcomm.2020.101262.

[2] D.J. Frisch, M. J.; Trucks, G. W.; Schlegel, H. B.; Scuseria, G. E.; Robb, M. A.; Cheeseman, J. R.; Scalmani, G.; Barone, V.; Mennucci, B.; Petersson, G. A.; Nakatsuji, H.; Caricato, M.; Li, X.; Hratchian, H. P.; Izmaylov, A. F.; Bloino, J.; Zheng, G.; Sonnenb, Gaussian 09, Revision D.01; Gaussian Inc., Wallingford, CT, (2013).

[3] M. Talaikis, O. Eicher-Lorka, G. Valinčius, G. Niaura, Water-induced structural changes in the membrane-anchoring monolayers revealed by isotope-edited SERS, J. Phys. Chem. C. 120 (2016) 22489–22499. https://doi.org/10.1021/acs.jpcc.6b07686.
